# Interaction of Arginine-Rich Surfactant-like Peptide Nanotubes with Liposomes

**DOI:** 10.1021/acs.biomac.4c01072

**Published:** 2024-10-29

**Authors:** Valeria Castelletto, Jani Seitsonen, Lucas R. de Mello, Ian W. Hamley

**Affiliations:** † School of Chemistry, Food Biosciences and Pharmacy, University of Reading, Whiteknights, Reading RG6 6AD, U.K.; ‡ Nanomicroscopy Center, Aalto University, Puumiehenkuja 2, FIN-02150 Espoo, Finland

## Abstract

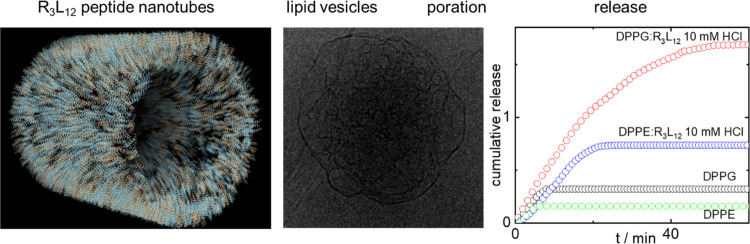

The interaction of the surfactant-like peptide (SLP) R_3_L_12_ bearing three cationic arginine residues with model liposomes is investigated in aqueous solution at various pH values, under conditions for which the SLP self-assembles into nanotubes. The structure of liposomes of model anionic lipid DPPG [1,2-dipalmitoyl-*sn*-glycero-3-phospho-rac-(1-glycerol)], or zwitterionic lipid DPPE [1,2-dipalmitoyl-*sn*-glycero-3-phosphoethanolamine] is probed using small-angle X-ray scattering and cryogenic-transmission electron microscopy. The unilamellar vesicles of DPPG are significantly restructured in the presence of R_3_L_12_, especially at low pH, and multilamellar vesicles of DPPE are also restructured under these conditions. The SLP promotes the release of cargo encapsulated in the vesicles as probed by calcein fluorescence, with notably higher release for anionic DPPG vesicles. Laurdan fluorescence experiments to probe membrane fluidity (lipid chain ordering) show that R_3_L_12_ destabilizes the lipid gel phase, especially for anionic DPPG. This model nanotube-forming SLP has promise as a pH-sensitive release system for vesicle-encapsulated cargo.

## Introduction

The linkage of hydrophobic and hydrophilic sequences in peptides or peptide conjugates can lead to amphiphilicity able to drive self-assembly into distinct nanostructures. One example is lipopeptides (also known as peptide amphiphiles, PAs) that have been of recent interest due to important applications in peptide therapeutics (for example lipidated peptide hormone derivatives for obesity or diabetes treatments) as well as a diversity of other potential uses.^1−3^ An additional class of amphiphilic peptide has been termed surfactant-like peptide (SLP).^4−9^ Such molecules comprise blocky sequences of hydrophobic and hydrophilic residues, typically a surfactant like “diblock” structure with a sequence of aliphatic hydrophobic residues such as alanine, leucine or isoleucine forming the “surfactant tail” and charged hydrophilic residues forming the “surfactant headgroup”. Charged residues among the natural amino acids comprise the basic residues arginine (R), lysine (K), and histidine (H) or the acidic residues aspartic acid (D) and glutamic acid (E).

Our group and others have been investigating the nanostructures of a diversity of SLPs especially those with cationic headgroups for which applications have been demonstrated including activities as antimicrobials and cell-penetrating peptides (CPPs).^10−22^ Surfactant-like peptides containing cationic residues are known to interact with oppositely charged (anionic) bacterial membranes causing their disruption, which is the main mode of activity. Via the same mechanism, they may have cell-penetration properties. We studied the self-assembly and antimicrobial activity of a series of SLPs containing terminal arginine residues. The SLP A_6_R forms nanosheets (or nanofibrils, depending on terminal capping) and has antimicrobial activity.^10,11^ The uncapped A_6_R assembles into nanotapes, which preferentially interact with lipid POPG in POPG/POPE vesicles [POPG = 2-oleoyl-1-palmitoyl-*sn*-glycero-3-phospho-rac-(1-glycerol), POPE = 2-oleoyl-1-palmitoyl-*sn*-glycero-3-phosphoethanolamine].^12^ Selective activity against Gram positive *Listeria monocytogenes* was observed for the capped A_6_R variant, whereas the uncapped version showed greater activity against this bacteria and also *Escherichia coli* and *Staphylococcus aureus*.^12^ The “bola-amphiphilic” SLP RA_3_R, containing arginine on both termini, was found to assemble into a polyproline II helix in water, which interacted strongly with the charged phosphoglycerol (PG) lipid POPG in lipid mixed vesicles^13^ It also restructures zwitterionic phosphocholine (PC) lipid vesicles. Our group also studied the interaction of arginine-rich SLPs R_4_F_4_ and R_3_F_3_ with mixed anionic/zwitterionic (PG/PE, PE: phosphoethanolamine) vesicles, and antimicrobial activity.^14^ Particularly pronounced activity against *Pseudomonas* species was found for R_4_F_4_, which also acts against *Pseudomonas aeruginosa* biofilms.^14^ The alanine-based SLPs we have studied generally form β-sheet fibrillar structures under appropriate conditions.

Among work from other groups on SLP self-assembly and interactions with membranes, Chen et al. investigated the self-assembly of three SLPs, A_3_K, A_6_K, and A_9_K, which showed distinct morphologies.^15^ The antimicrobial properties were found to be dependent on the length of the hydrophobic chain, peptide A_9_K being most active against *E. coli* and *S. aureus*, and also strongly disrupting the structure of anionic vesicles (of PG lipids) used as a model system for bacterial cell membranes. Lu, Xu and co-workers also examined the self-assembly of leucine-based SLPs such as (capped) L_4_K which forms different structures depending on the sequence of leucine enantiomers (d- or l- residues).^16^ In another example, they investigated the influence of amino acid chirality in SLP I_3_K variants on the handedness of twisted fibrillar superstructures.^17^ They also showed morphology control by changing the terminal residues in isoleucine peptide bola-amphiphiles such as KI_4_K, RI_4_R or HI_4_H.^18^ This team has also shown the highly selective antibacterial activity of SLPs such as A_9_K_2_ against both Gram-negative and -positive bacteria.^19^ Wong and co-workers have shown that arginine-rich peptides such as SLP R_6_W can act as cell-penetrating peptides (CPPs) able to drive slow leakage (of encapsulated dye) from mixed lipid vesicles.^20^ They describe the mechanism by which the arginine guanidinium group forms bidentate hydrogen bonds with phosphate groups on lipid headgroups, and have reviewed arginine-rich CPPs.^21^ The interaction of SLPs with model lipid (monoolein) membranes has been investigated for A_6_D, DA_6_, A_6_K, and KA_6_ via SAXS which confirms the induction of membrane curvature, and the formation of nonlamellar phases (including bicontinuous cubic phases) under appropriate conditions.^22^ Membrane fusion induced by coiled coil peptides is another important example of peptide-membrane interactions of relevance to viral infection by fusion of the viral coat with cell membranes.^23−25^ Short peptides able to form transmembrane structures such as ion channels via coiled coil assembly have also been reported.^26−29^

As part of our research program on bioactive self-assembling peptides, we have studied the self-assembly and conformation of SLP R_3_L_12_,^30,31^ which contains an oligomeric dodecyl leucine repeat, expected to promote α-helix formation.^32^ We showed via cryogenic-transmission electron microscopy (cryo-TEM) and small-angle X-ray scattering (SAXS) that this SLP forms nanotubes under suitable pH conditions (or tubular networks at very low pH = 1^30^ or globular structures at very high pH = 13^31^). The nanotubes contain peptide in α-helical conformation, confirmed by circular dichroism (CD) spectroscopy and wide-angle X-ray scattering. SAXS was used to determine the nanotube wall thickness (approximately 3 nm) which led to a model of the nanotube structure with a wall comprising a fully interdigitated bilayer of opposed R_3_L_12_ molecules with the arginine residues decorating the nanotube inner and outer surfaces and an inner hydrophobic leucine core within the nanotube wall.^30^ This represents a unique “cross-α” nanotube structure,^30^ that may be contrasted with other nanotube structures from channels within coiled coil packings of longer α-helical peptide with characteristic heptad repeats.^33−37^ In contrast to R_3_L_12_ the homologue K_3_L_12_ shows pH-dependent α-helix formation, and a transition from α-helices to β-sheets upon annealing.^38^

Here, we investigate the interaction of R_3_L_12_ with model membranes made of negatively charged lipid DPPG [1,2-dipalmitoyl-*sn*-glycero-3-phospho-rac-(1-glycerol)], or zwitterionic lipid DPPE [1,2-dipalmitoyl-*sn*-glycero-3-phosphoethanolamine]. The length of R_3_L_12_, like homologue K_3_L_12_^38^ is approximately matched to the thickness of a lipid bilayer and so it may anchor with, or insert into, the lipid membranes. Our aim was to investigate whether R_3_L_12_ can interact with and disrupt or penetrate lipid membrane and if so, to potentially use this as a triggerable release system for vesicle-encapsulated cargo. This is in fact demonstrated herein.

We investigated interaction of R_3_L_12_ with DPPG or DPPE membranes using several experimental techniques. SAXS, electron microscopy and Laurdan fluorescence were used to investigate the membrane structure, complemented by CD studies of peptide conformation in the presence of membranes. Electrophoretic mobility is used to show the influence of interactions with the peptide on vesicle charge. Release of encapsulated hydrophobic cargo within DPPG or DPPE vesicles was examined by calcein fluorescence experiments.

## Experimental Section

### Materials

Peptide R_3_L_12_ was supplied by Peptide Protein Research Ltd. (Fareham, United Kingdom) and is capped at both termini with acetyl at the N terminus and amide at the C terminus. The purity was >95% by high-performance liquid chromatography (HPLC) using an acetonitrile (0.1% TFA)/water (0.1% TFA) gradient. The molar mass by electrospray ionization mass spectrometry (ESI-MS) was 1885.525 gmol^–1^. DPPG, molar mass 744.95 gmol^–1^, and DPPE, molar mass 691.96 gmol^–1^, were obtained from Sigma-Aldrich. Scheme S1 shows the molecular structure of R_3_L_12_, DPPG and DPPE. In water, DPPG is anionic at neutral pH and DPPE is zwitterionic at acidic and neutral pHs.

In this work we study DPPG and DPPE samples at pH 7, 3 or 2. Phosphoglycerol lipids have a phosphate group p*K*_a_ around p*K*_a_ = 3^39^ so the degree of ionization is expected to be low at pH 2 and pH 3 (essentially zero at the lower pH) and the charge will be −1 at pH 7. Phosphoethanolamine lipids have p*K*_a_ values at around pH 1.7 (phosphate group) and 9.8–11.25 (amine group)^39,40^ and therefore DPPE is expected to be zwitterionic at pH 7, 3 or 2.

The calculated isoelectric point for R_3_L_12_ (capped at both termini) is pH 12.4, with a net charge +3 for pH < 10.

### Preparation of R_3_L_12_ Solutions

A stock solution of peptide was prepared by dissolving the peptide at 12 wt % R_3_L_12_ in hexafluoroisopropanol (HFIP), because R_3_L_12_ is a highly hydrophobic peptide. An aliquot of 1 μL of 12 wt % R_3_L_12_ in HFIP was added to 15 μL of ultrapure water inside a 1.5 mL Eppendorf. The Eppendorf was then vigorously vortexed while adding 2 × 145 μL of ultrapure water, 10 mM HCl or 100 mM HCl to obtain 0.04 wt % R_3_L_12_ at pH 4 (native), 2 or 1 respectively.

### Liposome Preparation

Liposome vesicles were prepared using the thin-layer hydration method. Weighed quantities of DPPG and DPPE were dissolved in chloroform, and thin lipid films were prepared as described previously.^41,42^ After this, lipid films were resuspended in water or in a 0.04 wt % R_3_L_12_ solution at pH 4 (native), 2 or 1. The lipid concentration of the resuspended films was 0.5 wt % for all the samples (0.5 wt % DPPG = 6.7 mM, 0.5 wt % DPPE = 7.2 mM). After resuspension, the solution was repeatedly heated to 65 °C (above the melting temperature of the lipids) and vortexed for 15 s. Finally, the solution was left to equilibrate for 1 h before experiments.

### Small Angle X-ray Scattering (SAXS) and Wide-Angle X-ray Scattering (WAXS)

SAXS data were collected on beamline B21 (Diamond Light Source, U.K.). Solutions were loaded into PCR tubes in an automated sample changer, and measurements were performed as described previously.^41^ Data were processed using ScÅtter and are presented as a function of wavenumber *q* = 4π sin θ/λ where θ is the scattering angle and λ is the X-ray wavelength.

WAXS experiments were carried out at beamline BM26^43^ at the ESRF (Grenoble, France). Solutions were manually loaded into a glass capillary with a 2 mm internal radius and measured using an X-ray beam with energy 12 keV. The WAXS signal was acquired with a Pilatus 300K-W (1472 × 195 pixels) detector with a pixel size of 172 μm × 172 μm. The scattering angular for the WAXS scattering angles were calibrated using Alumina (α-Al_2_O_3_). The scattered intensity recorded on area detectors was corrected by subtracting the scattering of a capillary containing water, before being reduced to the intensity as a function of scattering vector to obtain the one-dimensional (1D) intensity profiles using the software “bubble”,^44^ expressed in arbitrary units.

### SAXS Models

The SAXS curves for pure peptide solutions were fitted using a form factor describing the convolution of hollow cylinders (or long cylinders at high peptide concentration) with a set of Gaussian functions to represent the electron density variation across the nanotube wall, with an electron dense core and less dense surfaces.^10,45,46^ The fitting parameters for the cylindrical shell form factor were the core radius, *R*, the shell thickness, *D*_r_, and the scattering length density of the core, η_core_, shell, η_shell_, and solvent, η_solv_. The fitting parameter for the long cylinder form factor was the cylinder radius *R*_c_.^47^ The Gaussian bilayer form factor was originally formulated for a lipid bilayer membrane. The model assumes an electron density profile comprising Gaussian functions for the head groups on either side of the membrane and another Gaussian for the hydrocarbon chain interior. The midpoint of the bilayer is defined as *z* = 0 = *z*_C_.^48^ The model assumes a distribution of interhead group thicknesses 2*z*_H_, with an associated Gaussian width Δ(2*z*_H_). The fitting parameters of the model are the electron density of the headgroup, η_H_, the layer thickness, 2*z*_H_, the electron density of the hydrocarbon chains, η_H_, the standard deviation σ_H_ of the position of the Gaussian peak at *z*_H_, the standard deviation σ_C_ of the position of the Gaussian peak at *z*_C_,, and the Gaussian polydispersity in layer thickness, Δ(2*z*_H_).

The SAXS curves for pure liposomes or liposome/nanotube systems were fitted using the bilayer model described above. In addition, interactions between the bilayers were modeled using a Caillé structure factor with variables for the number of layers, *N*_1_, stacking separation, *d*, Caillé parameter, η, and scaling constant, *n*. All fitting was done using the software SASfit.^49,50^

### Cryo-Transmission Electron Microscopy (cryo-TEM)

Imaging was carried out using a field emission cryo-electron microscope (JEOL JEM-3200FSC), operating at 200 kV. Images were taken in bright field mode and using zero loss energy filtering (omega type) with a slit width of 20 eV. Micrographs were recorded using a Gatan Ultrascan 4000 CCD camera. The specimen temperature was maintained at −187 °C during the imaging. Vitrified specimens were prepared using an automated FEI Vitrobot device using Quantifoil 3.5/1 holey carbon copper grids with a hole size of 3.5 μm. Just prior to use, grids were plasma cleaned using a Gatan Solarus 9500 plasma cleaner and then transferred into the environmental chamber of a FEI Vitrobot at room temperature and 100% humidity. Thereafter 3 μL of sample solution was applied on the grid and it was blotted twice for 5 s and then vitrified in a 1/1 mixture of liquid ethane and propane at temperature of −180 °C. The grids with vitrified sample solution were maintained at liquid nitrogen temperature and then cryo-transferred to the microscope.

### Circular Dichroism (CD)

CD spectra were recorded using a Chirascan spectropolarimeter (Applied Photophysics, U.K.). Peptide solutions were placed in a parallel plaque cell (0.01 mm path length). Spectra were measured with a 0.5 nm step, 1 nm bandwidth, and 1 s collection time per step. The CD signal from the water background was subtracted from the CD data of the sample solutions. Data were smoothed using the instrument software with the default Savitzky–Golay filter, with the noise of the residual plot randomly distributed around zero to avoid any artificial distortion to the smoothed curve.

Ellipticity is reported as the mean residue ellipticity ([θ], in deg cm^2^ dmol^–1^) and calculated as:
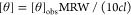
1[θ]_obs_ is the ellipticity measured in millidegrees, MRW is the mean residue molecular weight of the peptide (molecular weight divided by the number of amino acid residues = 15, Scheme S1), *c* is the total concentration in mg/mL, and *l* is the optical path length of the cell in cm.

The CD data was used to calculate the ratio [θ]_222_/[θ]_208_, a measure of coiled coil aggregation.^51,52^

### Dynamic Light Scattering (DLS)

DLS experiments were performed using an ALV/CGS-3 Compact Goniometer System with ALV/LSE-5003 correlator using vertically polarized incident light of wavelength 632.8 nm. Measurements were performed by placing the detector at 90° with repsect to the incident beam. The intensity autocorrelations functions were analyzed by the constrained regularized CONTIN method,^53^ to obtain distributions of hydrodynamic radius of the particle R_H_.

### ζ-Potential

The electrophoretic mobility (ζ-potential) was measured using a Zetasizer Nano ZS from Malvern Instruments. For experiments, 1 mL of sample was placed inside a disposable folded capillary cell. The sample was left to equilibrate for 120 s before measuring the ζ-potential, using an applied voltage of 50.0 V. The results presented are the average over three measurements. Solutions were prepared as in Table 1, and diluted 3 fold before ζ-potential experiments.

**Table 1:** Sample Compositions, pH and Nomenclature Used in This Work

composition	pH	name
0.04 wt % R_3_L_12_	4	R_3_L_12_ pH 4
0.5 wt % DPPG	7.19	DPPG pH 7
0.5 wt % DPPG:0.04 wt % R_3_L_12_	7.52	DPPG:R_3_L_12_ pH 7
0.5 wt % DPPE	7.34	DPPE pH 7
0.5 wt % DPPE:0.04 wt % R_3_L_12_	7.14	DPPE:R_3_L_12_ pH 7
0.04 wt % R_3_L_12_; 10 mM HCl	2	R_3_L_12_ pH 2
0.5 wt % DPPG; 10 mM HCl	3.14	DPPG pH 3
0.5 wt % DPPG:0.04 wt % R_3_L_12_; 10 mM HCl	3.09	DPPG:R_3_L_12_ pH 3
0.5 wt % DPPE; 10 mM HCl	2.53	DPPE pH 3
0.5 wt % DPPE:0.04 wt % R_3_L_12_; 10 mM HCl	2.73	DPPE:R_3_L_12_ pH 3
0.04 wt % R_3_L_12_; 100 mM HCl	1	R_3_L_12_ pH 1
0.5 wt % DPPG; 100 mM HCl	1.49	DPPG pH 2
0.5 wt % DPPG:0.04 wt % R_3_L_12_; 100 mM HCl	1.57	DPPG:R_3_L_12_ pH 2
0.5 wt % DPPE; 100 mM HCl	1.51	DPPE pH 2
0.5 wt % DPPE:0.04 wt % R_3_L_12_; 100 mM HCl	1.55	DPPE:R_3_L_12_ pH 2

### Fluorescence Spectroscopy

Experiments were carried out using a Varian Cary Eclipse spectrofluorometer. Solutions were loaded in a 10 mm light path quartz cell. Fluorescence experiments were run as part of the calcein and Laurdan assays described below.

For calcein assays, the solutions were first excited at λ_ex_ = 485 nm, and the fluorescence emission was measured from 510 to 670 nm. An emission fluorescence spectrum was obtained with a maximum intensity at λ_em_ = 544 nm. This information was used to run fluorescence kinetic experiments monitoring λ_em_ = 544 nm (λ_ex_ = 485 nm) over 60 min with a data acquisition interval of 0.1 s.

For Laurdan assays, the excitation spectra was first measured from 320 to 415 nm using λ_em_ = 440 nm. Then, the fluorescence emission spectra of Laurdan at 20 °C ≤ *T* ≤ 75 °C, was measured from 370 to 620 nm using λ_ex_ determined from the excitation spectra. The presented spectra were smoothed using the Savitzky-Golay method using software Origin.

### Calcein Fluorescence Assay

Calcein-loaded liposomes were prepared to study the release of calcein due to liposome disruption, in the presence of R_3_L_12_ nanotubes. The procedure is described as follows. Weighed quantities of DPPG and DPPE were dissolved in chloroform, and thin lipid films were prepared as described previously.^41,42^ After this, lipid films were resuspended in a solution of 0.6 wt % calcein at 16 mM NaOH. After resuspension, the solution was repeatedly heated at 65 °C and vortexed for 15 s, resulting in calcein-loaded DPPG or DPPE liposomes.

After liposome preparation, 200 mg of Sephadex G-50 Superfine column gel was put in a glass vial and covered with 2 mL of water. The mixture was left to stabilize and form a soft gel. A Pasteur pipette was used to build a column for the Sephadex G-50 Superfine gel. A small cotton ball was placed at the narrow end of the pipette, to filter spurious particles from the final product, while the narrow tip of the pipette was shortened to facilitate the release of the filtered solution. Finally, the Sephadex G-50 Superfine gel was put in the body of the pipette with the help of a spatula, attaining a 3 cm column height.

DPPG- or DPPE- calcein loaded liposome solutions were run through the Sephadex G-50 column by gravitational flow. The column and solutions were protected from the light by covering with aluminum foil during the period of the run. Solutions free of excess calcein were obtained from the filtration.

The size of the filtered DPPG or DPPE calcein- loaded liposomes was confirmed by DLS experiments. The fluorescence of calcein in such liposomes (λ_ex_ = 485 nm) gave a single peak at λ_em_ = 544 nm, with intensity *I*_0_. Then, 340 μL of 0.04 wt % R_3_L_12_ pH 2 was injected into 800 μL of calcein-loaded DPPG or DPPE liposomes. The intensity of the emission fluorescence at time *t*, *I_t_* (λ_ex_ = 485 nm, λ_em_ = 544 nm) was then measured during 60 min (data acquisition interval time: 0.1 s).

Afterward, complete DPPG or DPPE liposome lysis was induced by adding sodium dodecyl sulfate (SDS) to a final concentration of 2.5 wt % SDS. The intensity of the fluorescence emission of the solution at λ_em_ = 544 nm, *I*_max_, was then measured using λ_ex_ = 485 nm.

The experimental results were used to calculate the fractional permeabilization of the DPPE or DPPG liposomes as a function of time *f*_c_(*t*):
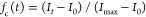
2*f*_c_(*t*) data was then used to calculate the cumulative release from liposomes, *R*(*t*) defined as:
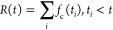
3A control experiment was also run for pure calcein-loaded DPPG or DPPE liposomes, in the absence of 0.04 wt % R_3_L_12_ pH 2.

For the dosing assay, calcein- loaded DPPG or DPPE liposomes were prepared and filtered as described above. Then 340 μL of 0.02, 0.04, 0.06, 0.08 or 0.1 wt % R_3_L_12_ pH 2 were injected into 800 μL of calcein- loaded DPPG or DPPE. The absorbance at λ = 544 nm was measured, using a Nanodrop instrument, at *t* = 0 (*A*_0_) and *t* = 60 min (*A*_60_). Then, liposome lysis was achieved by adding SDS to a final concentration of 2.5 wt % SDS. The absorbance at λ = 544 nm (*A*_m_) was measured following lysis. The fractional release was calculated as a function of the injected R_3_L_12_ concentration:
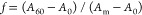
4

### Laurdan Fluorescence

Laurdan- loaded DPPG or DPPE liposomes were prepared using the thin-layer hydration method described above. Aliquots 1 mg of DPPG or DPPE were dissolved in 126 μL of 8.5 × 10^–4^ wt % Laurdan in chloroform. The chloroform was evaporated under a nitrogen stream to obtain a film, which was dried under vacuum for 2 h. The film was resuspended in 200 μL of the peptide solution, or the solvent used to prepare the peptide solution, to have 0.5 wt % lipid in 5 × 10^–4^ wt % Laurdan. After resuspension, the solution was repeatedly heated to 65 °C and vortexed for 15 s. Afterward, the solutions were left to rest for 1 h and diluted 5-fold before experiments.

All samples listed in Table 1 were loaded with laurdan, but some of them precipitated. In this work we present Laurdan fluorescence results obtained only for homogeneous samples.

The Laurdan fluorescence excitation spectrum was measured using λ_em_ = 440 nm. Afterward the fluorescence emission spectrum was measured as a function of the temperature, for 20 °C ≤ *T* ≤ 75 °C, using λ_ex_ = 356 or 358 nm, as determined from the fluorescence excitation spectra.

The generalized polarization factor of the Laurdan fluorescence, *g*, was then calculated as a function of the temperature *T* according to:^54−56^
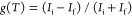
5where, *I*_i_ and *I*_f_ are the intensity of the fluorescence measured at the wavelength of the maximum intensity at 20 and 75 °C respectively. The dependence of *g* on *T* was used to determine the conformation of the lipidic chains.

## Results and Discussion

Arginine-based peptides can interact with lipid membranes and act as cell-penetrating peptides. Similarly, α-helical peptides can interact with and insert into lipid membranes as coiled coil bundles that can form channels in membranes.

Here, we investigate the interaction of the short α-helical SLP R_3_L_12_ with model anionic or zwitterionic lipid membranes in vesicles. As mentioned above, the length of R_3_L_12_ is close to that of a lipid bilayer which may facilitate its interaction with the membrane, although this will also be affected by the charge distribution on the self-assembled peptide nanostructure (here arginine-coated nanotubes, i.e., structures with a cationic exterior).

Here, DPPG or DPPE lipid films were resuspended in solutions containing short nanotubes (R_3_L_12_ pH 4), long nanotubes (R_3_L_12_ pH 2) or a nanotube network (R_3_L_12_ pH 1).^30^ The nanotubes are made of α-helical SLP R_3_L_12_, which is positively charged at the pH values studied in this work (pH < 10). The resuspension of the lipids in water gives liposomes, although we anticipated that this self-assembly process might be modified by the presence of peptide nanotubes in solution.

Table 1 gives a full list of the samples studied in this work, together with the corresponding pH and sample nomenclature (we use single digit notation of pH for convenience, the samples labeled pH 2 have pH 1.5 for instance). Samples DPPE pH 7 and DPPE pH 3 showed phase separation (SI Figure S1) and will not be considered further. From Table 1 it is clear that peptide:lipid samples have a pH similar to the pH of the corresponding pure lipid solution. For example, 0.04 wt % R_3_L_12_ has pH 4, but 0.5 wt % DPPG: 0.04 wt % R_3_L_12_ has pH 7.52, which is very similar to pH 7.19 measured for 0.5wt % DPPG.

As mentioned in the Experimental Section, the net charge on DPPG is expected to be low at pH 2 and pH 3, but it has negative charge −1 at pH 7. DPPE is expected to be zwitterionic (net charge 0) at pH 7, 3 or 2. The net charge of peptide R_3_L_12_ is +3 under all these conditions of pH. Thus, the strength of electrostatic interactions between the peptide and the lipid in samples in Table 1 is controlled by the pH of the solution.

In the following, we investigate the interaction of R_3_L_12_ with lipid membranes via SAXS/WAXS and electron microscopy, complemented by CD studies of peptide conformation in the presence of membranes. We use electrophoretic mobility to show the influence of interactions with the peptide on vesicle charge. Release of encapsulated hydrophobic cargo within the vesicles is examined by calcein fluorescence experiments. Finally, we perform Laurdan fluorescence experiments to study the organization of lipidic chains in the presence of R_3_L_12_.

**Figure 1:**
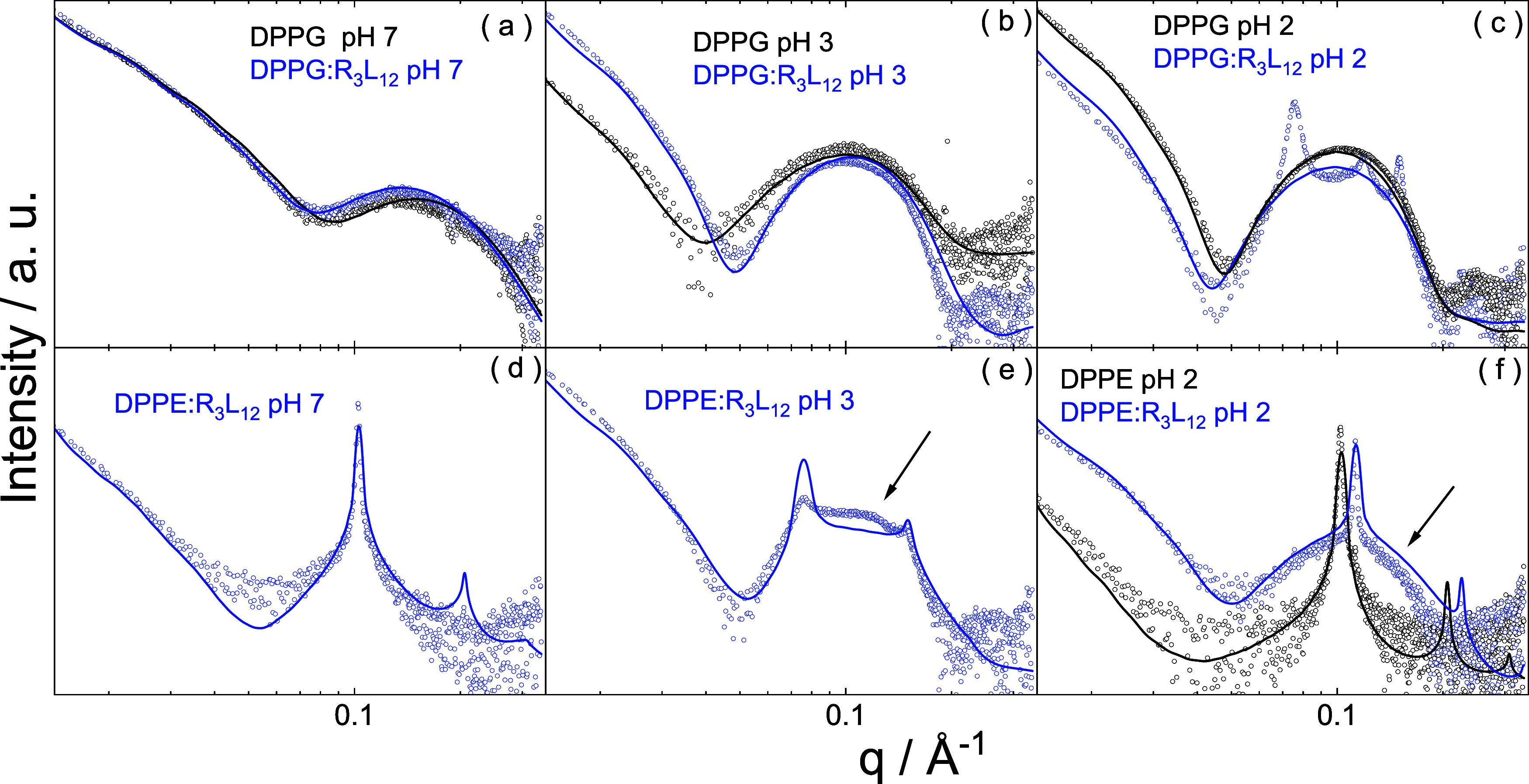
SAXS data for DPPG or DPPG:R_3_L_12_ at (a) pH 7, (b) pH 3 or (c) pH 2. SAXS data for DPPE:R_3_L_12_ at (d) pH 7, (e) pH 3 and (f) pH 2 (also DPPE). The full lines are the fits to the experimental data. The parameters extracted from the fitting are listed in SI Tables S2 and S3. The arrows indicate diffuse scattering.

The influence of the peptide on the lipid membranes was first examined using SAXS and WAXS. The corresponding data are shown in Figure 1 and SI Figures S2–S6.

SAXS data for the R_3_L_12_ nanotubes (solutions without lipids) used to prepare samples in Table 1, is presented in SI Figure S2 for reference, with fit parameters listed in SI Table S1. SAXS data for a R_3_L_12_ dilution series, used for the dosing assay described below, was also obtained and is presented in SI Figure S3, with fit parameters listed in SI Table S2. The obtained nanotube radius and wall thickness are in satisfactory agreement with our previously reported values.^30^ To test the stability of the nanotubes to the heat/vortex treatment to prepare peptide/liposome mixtures, SAXS was performed on the peptide before and after this procedure. The data shown in SI Figure S4 confirm that the nanotubes are stable under such treatment.

The SAXS data for the mixtures of the different peptide nanotube mixtures with the liposomes is displayed in Figure 1 (a detail of Figure 1c is given in SI Figure S5). The SAXS data was fitted using models for Gaussian bilayer form factor with multilamellar structure factors as needed, as detailed in the Experimental Section. The parameters extracted from the SAXS fitting for samples containing DPPG or DPPE liposomes are listed in SI Tables S3a and S3b respectively. The SAXS data shows features from vesicle structures and there is no signature of peptide nanotubes (in contrast to the data in SI Figure S2). Together with the fact that the SAXS features from vesicle structure are altered in the presence of peptide, this shows that the self-assembly of R_3_L_12_ is influenced by the presence of the liposomes and the changes in the vesicle SAXS profiles indicate that peptide restructures vesicles, likely due to incorporation of the peptide in the vesicles.

SAXS data in Figure 1a–c show that DPPG and DPPG:R_3_L_12_ pH 7 or pH 3 together with DPPG pH 2 form unilamellar vesicles, since the SAXS intensity profile corresponds to a bilayer form factor and there are no Bragg peaks associated with bilayer repeats. We previously reported the unilamellar form factor of DPPG liposomes themselves.^42^ SAXS data in Figure 1a–c show that there are there are differences in the shape of the form factor of the unilamellar structure; in particular fit parameters show a notable increase in bilayer spacing for DPPG:R_3_L_12_ pH 3 (SI Table S3a). In contrast, DPPG:R_3_L_12_ pH 2 solutions present the coexistence of two lamellar structures with cell parameters *d* = 52.1 Å and *d* = 83.2 Å respectively (Figure 1c, further detail in SI Figure S5). The former spacing is close to the DPPG lamellar L_β′_ phase (stable near room temperature) bilayer spacing *d* = 57.9 Å,^57,58^ The coexisting peaks suggest the possible coexistence of pure DPPG vesicles and vesicles with expanded bilayer spacings that are likely to incorporate R_3_L_12_.

SAXS data in Figure 1d–f show that, in contrast to DPPG, DPPE forms multilamellar vesicles in the presence of R_3_L_12_. The range of the lamellar order (as characterized by the fit parameter N_1_ listed in SI Table S3b) decreases for DPPE:R_3_L_12_ pH 3 and is higher for for DPPE:R_3_L_12_ pH 2 where a nanotube network was used to resuspend the lipid film. A notable feature of the SAXS data for the DPPE vesicles in the presence of R_3_L_12_ is the development of pronounced diffuse scattering features at the base of the principal lamellar Bragg peak (Figure 1d–f) and additionally between the Bragg peaks for the pH 2 sample (Figure 1e). Such strong diffuse scattering is a signal of strong layer fluctuations or perforations.^59^ The data in Figure 1e with an additional broad peak between the lamellar reflections as well as broad diffuse scattering at the base of the Bragg reflections in particular suggests that the lamellae (bilayers) may be perforated.^59^ This was also confirmed by cryo-TEM to be discussed shortly. The measured lamellar L_β_ phase *d*-spacing value *d* = 61 Å (SI Table S3b) for DPPE is in good agreement with the previously reported value (room temperature, hydrated sample) *d* = 63 Å.^60^

The WAXS data corresponding to the SAXS data presented in Figure 1 are shown in SI Figure S6. The WAXS peak position was used to calculate the lateral spacing *d*_l_ = 2π/*q*_0_ (*q*_o_ = wavenumber for the peak maximum) between lipid chains. Calculated *d*_l_ values are displayed in the figure caption of SI Figure S6. The WAXS profile for DPPG (SI Figure S6a) is similar to that reported previously, for which the asymmetric peak shape was decomposed into peaks from a two-dimensional (2D) orthorhombic unit cell of lipid chains in the L_β′_ phase.^57^ The WAXS data for DPPE and mixtures show a higher degree of order than for DPPG with several sharp peaks (indexed in SI Figure S6), although previously only one WAXS peak was reported for hydrated DPPE at room temperature.^60^ The WAXS data in SI Figure S6 show that, on the whole, the lateral distance between lipid chains is not affected by the addition of peptide to the liposomes, which suggests that the peptide helices do not mix homogeneously with the lipid chains in the bilayer.

The surface charge of the liposomes was analyzed by measuring the electrophoretic mobility (ζ-potential) for the samples in Table 1, the values being listed in SI Table S4. Since it contains three cationic arginine residues, R_3_L_12_ is positively charged (+3 charge for the capped peptide below the arginine p*K*_a_ value) and so a positive ζ-potential is measured in solutions from pure peptide (footnote in Table S4). DPPG liposomes are negatively charged, with a negative ζ-potential in solution which becomes less negative upon addition of peptide nanotube solution (SI Table S4). For DPPE liposomes all ζ-potential values are positive because DPPE is a zwitterionic lipid. Indeed, the absolute value of the ζ-potential changes upon addition of peptide, but its sign remains unaltered, pointing to a redistribution of charge at the surface of the liposomes in the presence of R_3_L_12_.

The CD spectra measured for DPPG:R_3_L_12_ and DPPE:R_3_L_12_ samples are shown in SI Figure S7 together with the [θ]_222_/[θ]_208_ ratio calculated from the plotted data. The results confirm the formation of α-helical coiled coil structures for all solution conditions studied. Decreasing the pH decreases the population of coils in DPPG:R_3_L_12_ samples, but does not induce any substantial change in the secondary structure of DPPE:R_3_L_12_ samples.

**Figure 2:**
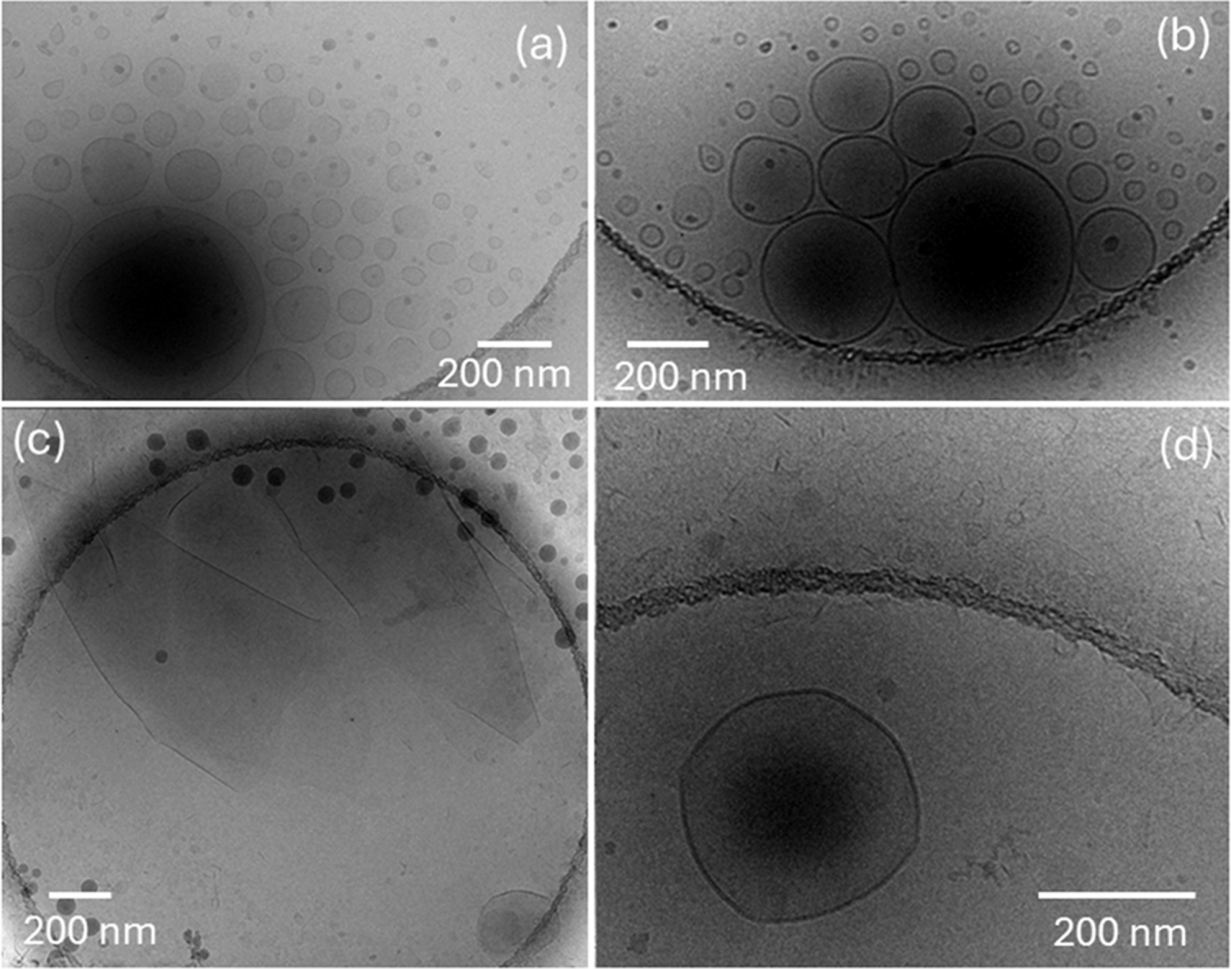
Cryo-TEM images for (a, b) DPPG:R_3_L_12_ pH 7 or (c, d) DPPE:R_3_L_12_ pH 7.

Cryo-TEM was used to image the vesicle structures and to probe the influence of peptide on the liposome morphology. Together with SAXS it provides a comprehensive characterization of vesicle restructuring induced by SLP R_3_L_12_. Cryo-TEM images for DPPG:R_3_L_12_ pH 7 and DPPE:R_3_L_12_ pH 7 solutions are shown in Figure 2a–d respectively. The images in Figure 2 display a similar population of polydisperse vesicles for both samples. The distribution of vesicle diameters measured for DPPG:R_3_L_12_ pH 7 and DPPE:R_3_L_12_ pH 7 solutions are displayed in the histograms in SI Figure S8a,b respectively. For both samples, although the vesicle diameter can reach 1300 nm, the highest population is for vesicles with diameter ∼100 nm. While the cryo-TEM images for DPPG:R_3_L_12_ pH 7 only show vesicles, the cryo-TEM images for DPPE:R_3_L_12_ pH 7 also show evidence for sheet-like structures (in Figure 2c) or some very short nanotubes (present in the background in Figure 2d), suggesting that R_3_L_12_ interacts more strongly with DPPG than with DPPE.

**Figure 3:**
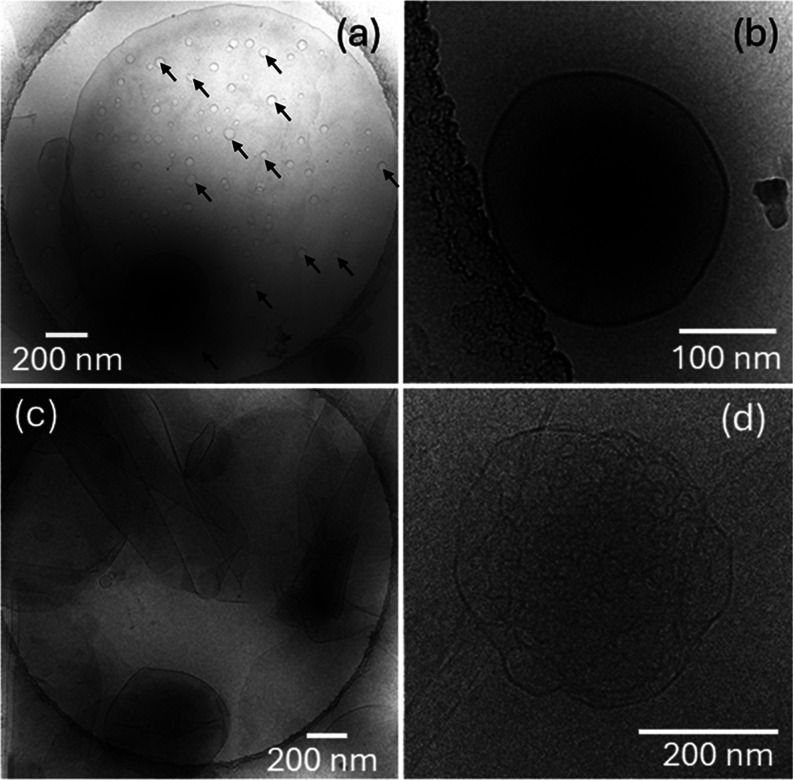
Cryo-TEM images for (a, b) DPPG:R_3_L_12_ pH 3 or (c, d) DPPE:R_3_L_12_ pH 3. The image in (d) suggests that long R_3_L_12_ nanotubes can restructure DPPE liposome walls. The arrows in (a) indicate small circular structures (adsorbed vesicles or nanosheet holes).

Cryo-TEM images for DPPG:R_3_L_12_ pH 3 and DPPE:R_3_L_12_ pH 3 solutions are shown in Figure 3a–d respectively and SI Figure S9. The distribution of vesicle diameters measured for DPPG:R_3_L_12_ pH 3 and DPPE:R_3_L_12_ pH 3 solutions are displayed in the histograms in Figure S8a,b respectively. For both samples, although the vesicle diameter can reach ∼1200 nm, they highest population is for vesicles with diameter ∼45 and 100 nm for DPPG:R_3_L_12_ pH 3 and DPPE:R_3_L_12_ pH 3 respectively.

The images in Figure 3a,b for DPPG:R_3_L_12_ pH 3 solution show vesicles coexisting with large plate-like sheets covered with smaller regular circular vesicles (or perforations in the sheets). In Figure 3c,d, in DPPE:R_3_L_12_ pH 3 solution, polydisperse vesicles coexist with extended floating sheets. Large plate-like sheets, covered with smaller regular circular vesicles were also observed for DPPE:R_3_L_12_ pH 3 (images not shown). In addition, some images suggest the insertion of some nanotubes in the liposome walls (Figures 3d and S9b,c). Equally interestingly, SI Figure S9a clearly shows that nanotubes can unfold into wide sheets. The insertion of a population of nanotubes into the vesicle walls may be responsible for the particularly strong diffuse scattering features observed in the SAXS data for the DPPE:R_3_L_12_ pH 3 solution (Figure 1e and associated discussion).

**Figure 4:**
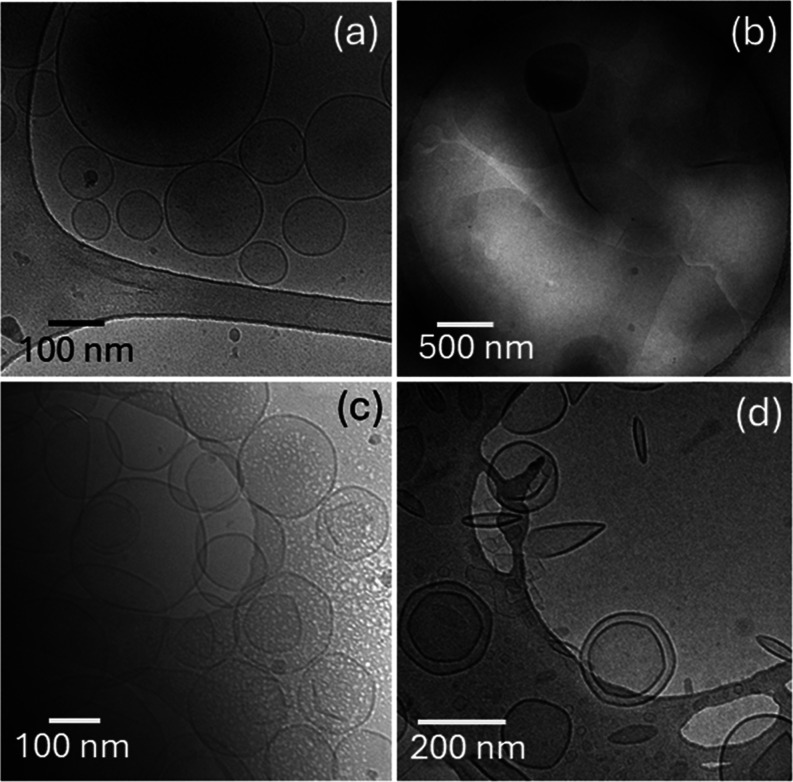
Cryo-TEM images for samples at pH 2: (a) DPPG, (b) DPPG:R_3_L_12_, (c) DPPE, (d) DPPE:R_3_L_12_.

Figure 4a,b show cryo-TEM images for DPPG pH 2 and DPPG:R_3_L_12_ pH 2 while Figure 4c,d show cryo-TEM images for DPPE pH 2 and DPPE:R_3_L_12_ pH 2. Sample DPPG pH 2 shows unilamellar vesicles (Figure 4a). The distribution of vesicle diameters is displayed in SI Figure S8a. In contrast, sample DPPG:R_3_L_12_ pH 2 (Figure 4b), consists mainly of large floating sheets with extremely scarce vesicles. In this case, the strong interaction between R_3_L_12_ and DPPG creates a self-assembly motif different from the nanotube network or the vesicle. Cryo-TEM images for DPPE pH 2 (Figure 4c) shows coexistence of unilamellar and multilamellar vesicles. Sample DPPE:R_3_L_12_ pH 2 (Figure 4d), consists of multilamellar vesicles coexisting with some excess R_3_L_12_ nanotube network and very short sheet-like structures. This suggests that a relatively weak DPPE-R_3_L_12_ interaction allows for the retention of the nanotube morphology of the peptide. The distribution of vesicle diameters measured for samples in Figure 4c,d is displayed in SI Figure S8b.

Dynamic light scattering (DLS) was used to determine changes in the liposome hydrodynamic radius *R*_H_ upon addition of peptide. The intensity-weight size distributions are displayed in SI Figure S10. The average *R*_H_ was estimated from the position of the maxima in the curves, and the distribution was quantified as Δ*R*_H_, the width at half height of the peaks. In general, *R*_H_ (SI Table S4) increases upon addition of peptide and the values are in good agreement with sizes measured by cryo-TEM (SI Figure S8).

As discussed above, cryo-TEM images for DPPG:R_3_L_12_ pH 3 and DPPE:R_3_L_12_ pH 3 show evidence for nanotubes interacting with vesicle walls (Figure 3d and SI Figure S9). This suggests that R_3_L_12_ nanotubes interact with vesicles and that this could be used as the basis of a controlled release system. We used the model fluorophore calcein, widely used in vesicle pore formation studies of the release of encapsulated cargo, to investigate this.

DLS size distributions measured for DPPG and DPPE liposomes loaded with calcein are shown in SI Figure S11a. Loading the liposomes with calcein, leads to a slight increase in *R*_H_ compared to *R*_H_ values listed in SI Table S4. As expected, the fluorescence emission of calcein can be measured for DPPG and DPPE loaded liposomes (SI Figure 11b, λ_em_ = 544 nm for λ_ex_ = 485 nm).

**Figure 5:**
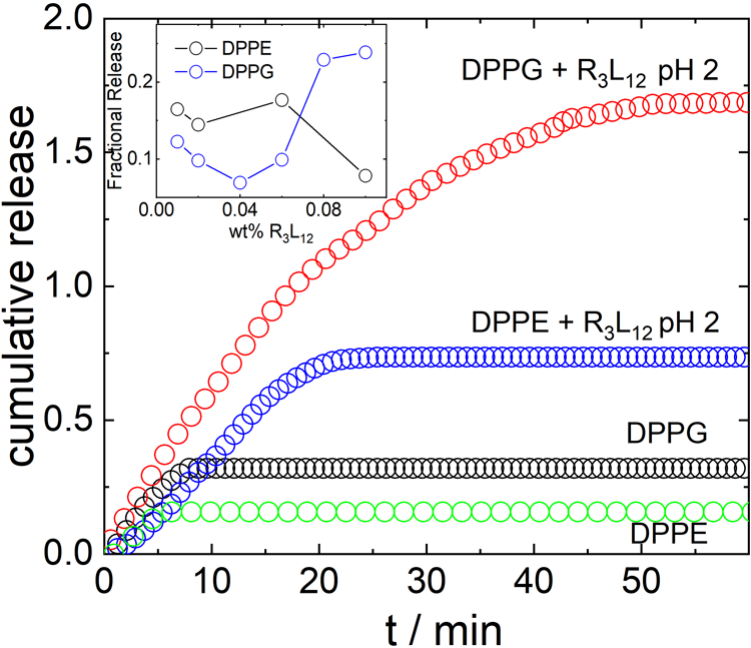
Cumulative release measured for the release of calcein encapsulated in (black circles) DPPG or (green circles) DPPE liposomes. Release of calcein encapsulated in (red circles) DPPG or (blue circles) DPPE liposomes, following the injection of R_3_L_12_ pH 2 long nanotubes in the solution. The inset shows the fractional release by DPPG or DPPE liposomes after 60 min incubation with R_3_L_12_ pH 2 solutions.

Figure 5 shows the cumulative permeability, *R* (eq 3) measured for the release of calcein encapsulated in DPPG or DPPE liposomes, following the injection of R_3_L_12_ pH 2 in the solution. This data is plotted together with that for a control (calcein encapsulated in DPPG or DPPE liposomes with no injection of R_3_L_12_ pH 2). The data show that there is a fast initial burst release of calcein from DPPE liposomes induced by the interaction of R_3_L_12_ with the lipid membrane and the total permeability is substantially higher than control (where a fraction of calcein is released at first from a population of “leaky” vesicles). The release of calcein by DPPG vesicles following R_3_L_12_ pH 4 injection is much higher than the release of calcein from DPPG vesicles alone. In addition, the release of calcein is much higher for DPPG than for DPPE vesicles injected with R_3_L_12_ pH 4 (Figure 5), proving that R_3_L_12_ interacts more strongly with anionic DPPG than with zwitterionic DPPE.

The inset in Figure 5 shows the results for the concentration-dependent fractional release of calcein measured 60 min after injecting calcein- loaded DPPG or DPPE liposomes with a R_3_L_12_ pH 2 solution. SI Figure S3 shows the SAXS data for the injected R_3_L_12_ pH 2 solutions, fitted as described in the Experimental Section. SAXS curves for 0.02–0.08 wt % R_3_L_12_ pH 2 were fitted to the form factor of peptide nanotubes, while data for 0.1 wt % R_3_L_12_ pH 2 was fitted to the form factor of a long cylinder. Fit parameters are listed in SI Table S2. The inset in Figure 5 shows that the fractional release increases with R_3_L_12_ pH 2 concentration only for calcein loaded DPPG liposomes, giving evidence of a strong interaction between the lipid and the peptide.

Laurdan fluorescence experiments were performed to study the fluidity of the lipid membranes in the presence of R_3_L_12_. SI Figures S12 and S13 display the emission fluorescence curves for the samples that formed homogeneous solutions. The excitation wavelength used for each curve is displayed in each figure caption, together with the wavelengths used to calculate the generalized polarization factor *g*(*T*) (eq 5).

**Figure 6:**
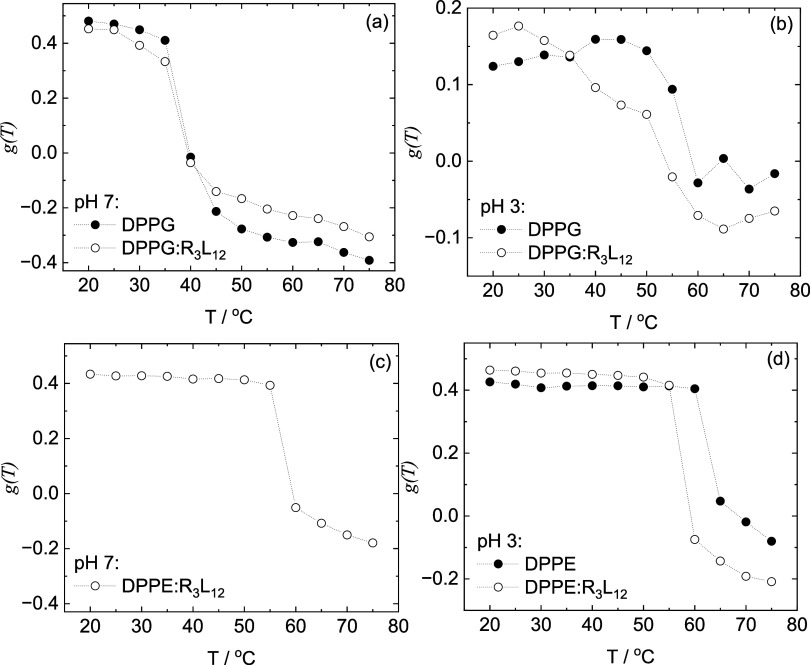
Laurdan generalized polarization factor calculated for Laurdan loaded liposomes: (a) DPPG and DPPG:R_3_L_12_ pH 7, (b) DPPG and DPPG:R_3_L_12_ pH 3, (c) DPPE:R_3_L_12_ pH 7, (d) DPPE and DPPE:R_3_L_12_ pH 3.

The spectra in SI Figure S12 were used to calculate *g*(*T*) for DPPG and DPPG:R_3_L_12_ pH 7 (Figure 6a) and pH 3 (Figure 6b). The spectra in SI Figure S13 were used to calculate *g*(*T*) for DPPE:R_3_L_12_ pH 7 (Figure 6c) and for DPPE and DPPE:R_3_L_12_ pH 3 (Figure 6d).

The lipid gel phase melting temperatures *T*_m_ for DPPG and DPPE have been reported in the literature as 40.6 °C (DPPG) and 64.1 °C (DPPE).^61^ In a previous work we reported 40.5 °C and 63.9 °C as *T*_m_ values for DPPG and DPPE respectively, based on DSC measurements.^42^

Laurdan undergoes a red spectral shift during the membrane phase transition from gel to fluid, which is attributed to dipole relaxation and the sensitivity to the polarity of the environment.^54,55^ Based on generalized polarization from Laurdan fluorescence, Figure 6a shows that for both DPPG and DPPG: R_3_L_12_ pH 7 the onset of the gel-to-fluid transition starts at 35 °C, and it is not influenced by the addition of R_3_L_12_ to the lipid membrane. Generalized polarization *g*(*T*) values for DPPG membranes at pH 3 (Figure 6b) are much lower than at pH 7 (Figure 6a), independently of the presence of peptide in the membrane, showing an intrinsic higher disorder of DPPG membranes at pH 3. The data in Figure 6b shows that for DPPG and DPPG:R_3_L_12_ pH 3 the onset of the gel-to-fluid transition starts at 50 and 35 °C respectively. At pH 3, the addition of R_3_L_12_ to the DPPG membrane reduces the order of the lipid chains lowering the value of *T*_m_ by ∼15 °C. Figure 6c shows that for DPPE:R_3_L_12_ pH 7 the onset of the transition starts at 55 °C. Figure 6d shows that for DPPE and DPPE:R_3_L_12_ pH 3 the onset of the transition starts at 65 and 60 °C respectively. At pH 3, the addition of R_3_L_12_ to the DPPE membrane reduces the order of the lipid chains lowering the value of *T*_m_ by ∼ 5 °C. The Laurdan fluorescence results thus indicate that addition of R_3_L_12_ causes a reduction in the gel-to-fluid transition temperature for both DPPG and DPPE at pH 3, pointing to the interaction of the peptide with the lipid membranes. The effect is significantly larger for anionic DPPG, highlighting the role of charge.

## Conclusions

Here SAXS has provided unique insights into the restructuring of lipid vesicles by a surfactant-like peptide and reveals the development of diffuse scattering features due to fluctuations or perforations of lamellae of DPPE. SAXS shows that SLP R_3_L_12_ interacts with and restructures unilamellar vesicles of anionic lipid DPPG in a pH-dependent fashion to produce populations of multilamellar vesicles with two different lamellar spacings at low pH. The DPPG vesicles retain a unilamellar structure upon adding R_3_L_12_ nanotubes at native pH or in 10 mM HCl. Unexpectedly, R_3_L_12_ also restructures multilamellar vesicles of zwitterionic lipid DPPE under suitable low pH conditions leading to changes in lamellar spacing and particularly notable enhancement of diffuse scattering. The diffuse scattering is associated with the formation of perforations in the lipid membranes, also suggested by cryo-TEM images.

Dynamic light scattering shows an increase in the average size of vesicles in the presence of R_3_L_12_, and in the case of DPPE in particular an increase in the width of the vesicle size distribution. This suggests that R_3_L_12_ may facilitate membrane fusion leading to larger vesicles. Fusion of zwitterionic lipid membranes has previously been reported for coiled-coil peptide pore formers and was found to be sensitive to bilayer hydrophobic layer properties, including the match between bilayer thickness and peptide size.^62^ As noted in the Introduction section, the length of R_3_L_12_ is closely matched to that of typical lipid membranes. However, the cationic charge of arginine, present at the surface of the nanotubes is unlikely to favor insertion of nanotubes into lipid membranes. The interaction of R_3_L_12_ with lipid vesicles is evidenced by changes in the lipid melting temperature revealed by laurdan fluorescence as well as influence on the ζ-potential values. WAXS also shows changes in lipid packing of DPPE in the presence of R_3_L_12_. CD shows that there is reduction in coiled coil ordering due to interaction of the peptide with with DPPG vesicles.

We demonstrated the successful selective release of calcein from vesicles induced by R_3_L_12_ membrane breakage. The release is significantly higher from DPPG vesicles than DPPE vesicles, which is ascribed to the electrostatic interaction between the cationic peptide and the anionic DPPG membrane. The release is observed for 0.21 mM R_3_L_12_ with 6.9 mM DPPG or 7.2 mM DPPE, i.e., at a molar ratio lipid: peptide of 1:35. It is important to contrast the two mechanisms relevant to our studies. In the first, a lipid film is resuspended with a SLP nanotube solution, and we show that liposomes are affected by the presence of the peptide during the resuspension treatment (temperature and agitation conditions applied) which may also influence nanotube assembly. In the second case, we study already-formed liposomes loaded with calcein or Laurdan, and show that they are restructured due to interaction with SLPs. In the latter case, the liposomes are exposed to well-structured nanotubes that interact with preformed membranes rather than during the assembly process.

**Figure 7:**
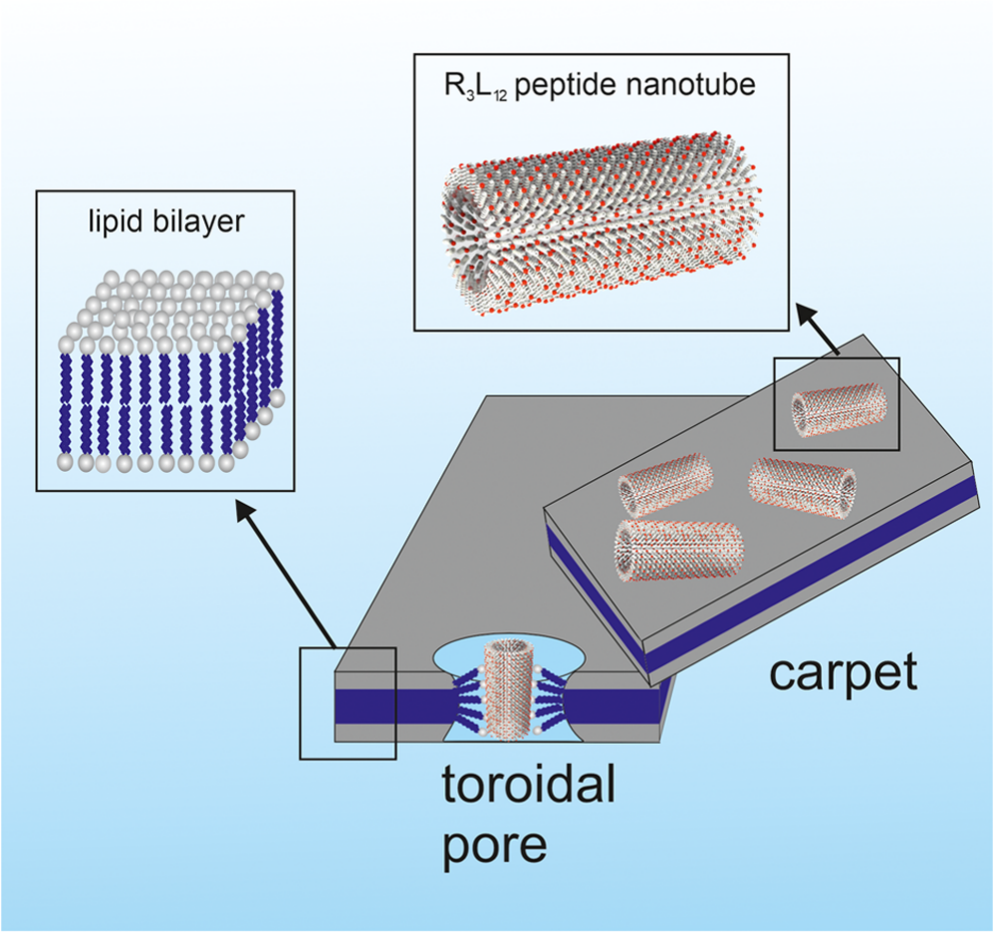
Schematic of one proposed mode of action. Arginine-coated nanotubes disrupt lipid membranes via a carpet-like model through interaction of the lateral cationic residues with charged membranes through electrostatic interactions or zwitterionic interactions through hydrogen bonding of guanidinium groups with lipid phosphate headgroups. Toroidal pores in the membrane may also be formed.

Since R_3_L_12_ nanotubes comprise arginine-coated nanotube walls with an oligo-leucine nanotube wall interior, we do not expect them to directly insert into the membrane in nanotube form due to the charged nature of the nanotube surfaces, which is not compatible with the hydrophobic interior of the lipid membrane. Instead, we propose that R_3_L_12_ breaks lipid membranes either through a “carpet-like” mechanism as sketched in Figure 7, or through toroidal pore formation in which the lipid headgroups form the surface of the pores Both these models are widely discussed as mechanisms for the activity of antimicrobial peptides.^63−66^ The electrostatic interaction between the arginine residues and anionic lipid headgroups of DPPG leads to breakage of the membrane. Alternatively, hydrogen bonding interactions between arginine guanidinium groups and zwitterionic lipid headgroups of DPPE may lead to membrane curvature (fluctuations) and/or perforations as previously reported for other arginine-rich peptides interacting with lipid membranes rich in zwitterionic lipids.^21,67^ It should be noted that it is likely that R_3_L_12_ interacts with lipid bilayers in monomeric form (molecules dissociated from nanotubes) in addition to deposition of intact nanotubes (Figure 7).

In summary, the surfactant-like peptide R_3_L_12_ that forms nanotubes under defined pH conditions (here acidic solutions) disrupts both anionic and zwitterionic membranes. This leads to release of cargo encapsulated in vesicles, which is more pronounced for anionic liposomes due to favorable electrostatic interactions.

## Supporting Information Available

Scheme showing molecular structures, images of samples in vials, additional SAXS data, CD spectra, additional cryo-TEM images and associated histogram analysis of vesicle diameters, hydrodynamic radius distributions from DLS, calcein and Laurdan fluorescence spectra, tables of SAXS fitting parameters and table of hydrodynamic radius and ζ-potential values (PDF)
