# Supplementary material for: Interaction of Arginine-Rich Surfactant-like Peptide Nanotubes with Liposomes
Source: Biomacromolecules. 2024 Oct 29;25(11):7410–20. doi: 10.1021/acs.biomac.4c01072 (PMC11558666; doi:10.1021/acs.biomac.4c01072)
Supplement: Supplementary file 1 — bm4c01072_si_001.pdf [file bm4c01072_si_001.pdf]

Supporting Information

Interaction of Arginine-Rich Surfactant-Like Peptide Nanotubes with Liposomes

Valeria Castelletto,[1] Jani Seitsonen,[2] Lucas R. de Mello[1], Ian W Hamley[1,*]

[1] *School of Chemistry, Food Biosciences and Pharmacy, University of Reading, Whiteknights, Reading RG6 6AD, U.K.*

[2] *Nanomicroscopy Center, Aalto University, Puumiehenkuja 2, FIN-02150 Espoo, Finland*

* Author for correspondence: I.W.Hamley@reading.ac.uk

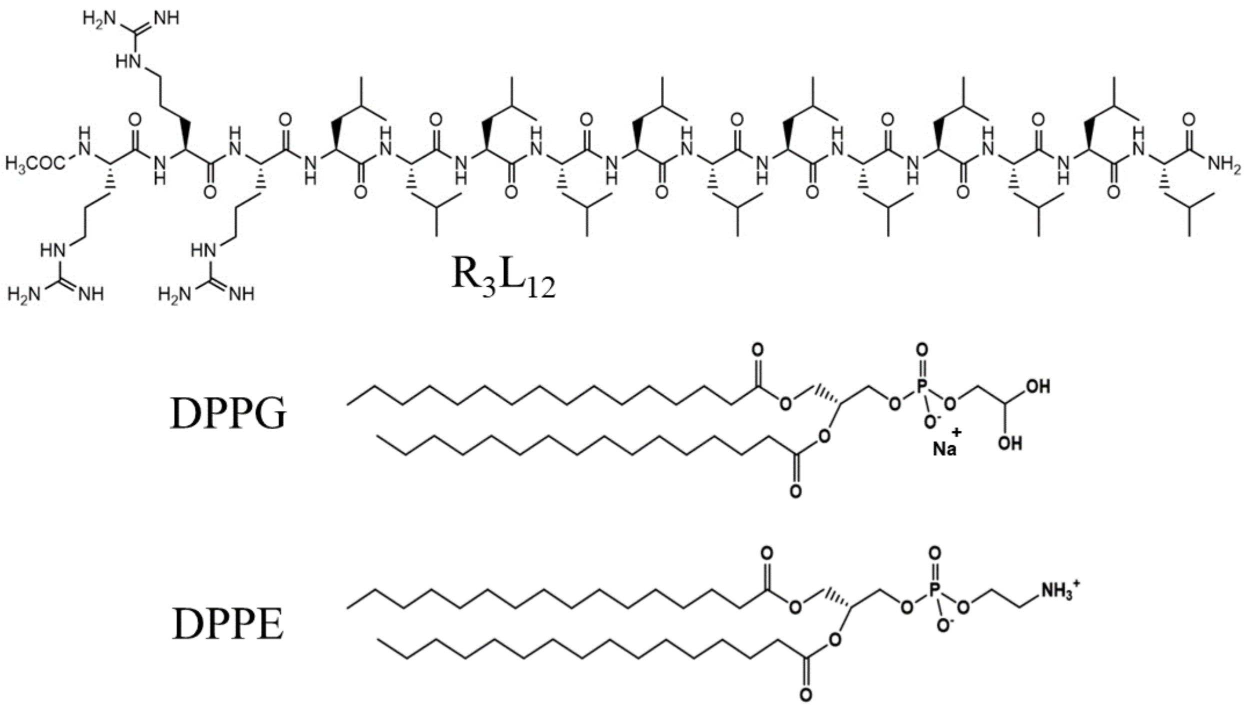

Scheme S1. Molecular structure of peptide R_3L_{12}, together with DPPG and DPPE lipids.

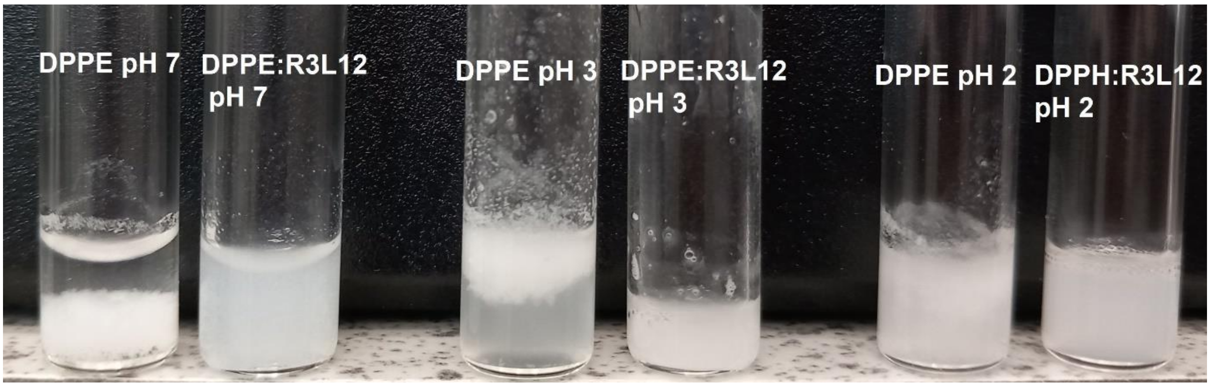

Figure S1. Vials showing DPPE or DPPE:R_3L_{12} solutions at different concentrations of HCl (Table 1). Phase separation can be observed for samples DPPE:R_3L_{12} pH 7 or pH 3.

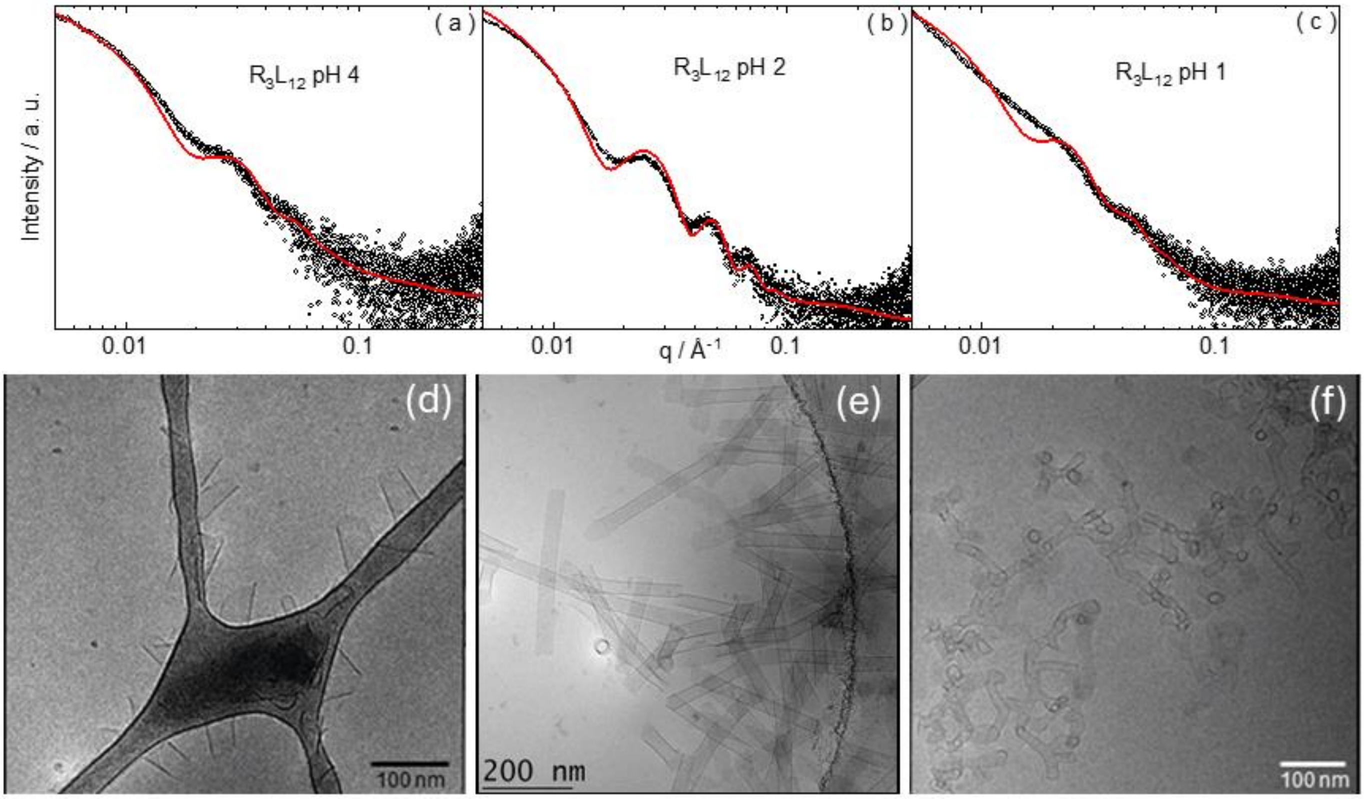

Figure S2. SAXS data measured for 0.04 wt% R_3L_{12} at (a) pH 4, (b) pH 2, (c) pH 1. The full lines are the fits to the experimental data. The parameters extracted from the fits are listed in Table S1. Cryo-TEM images for 0.04 wt% R_3L_{12} at (d) pH 4, (e) pH 2, (f) pH 1. The images in (d) and (f) are taken from ref.[1]

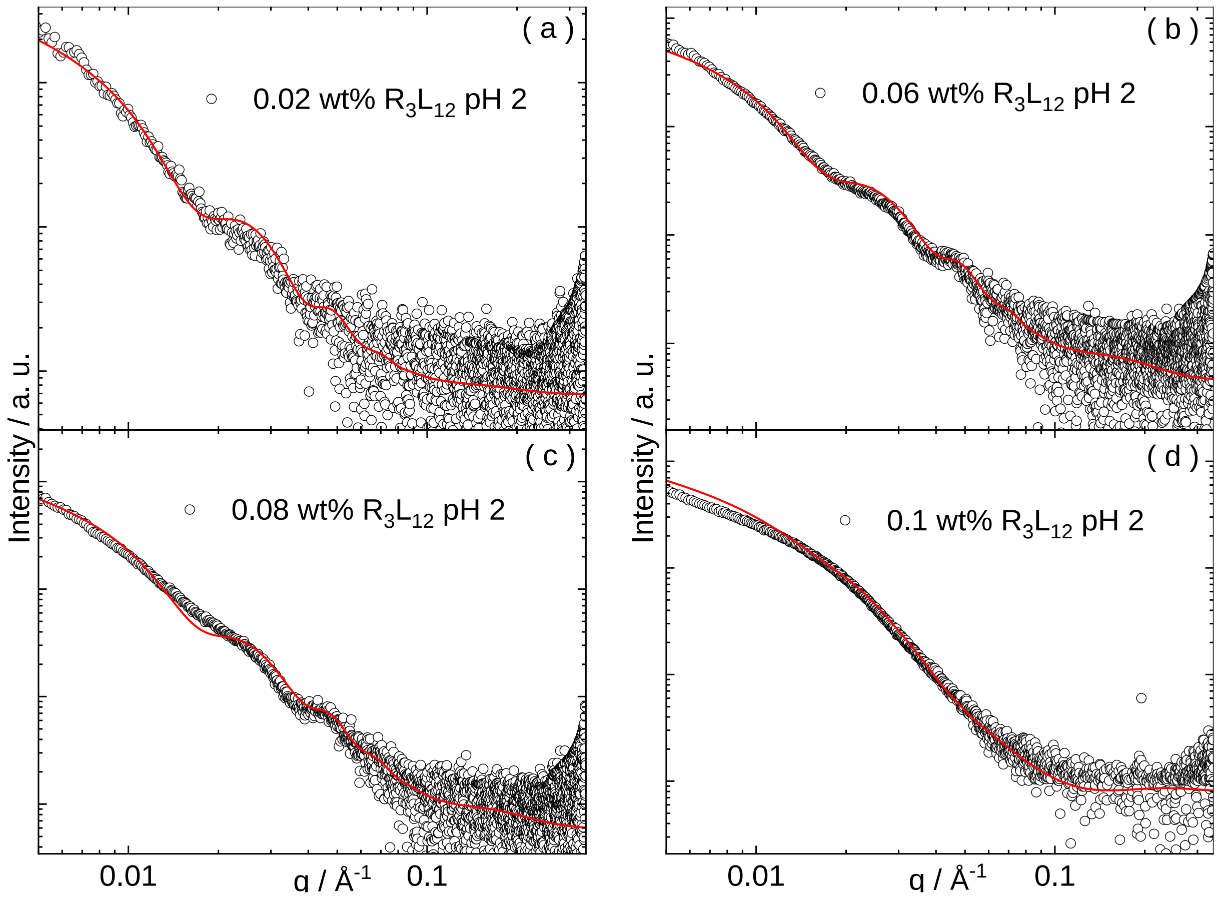

Figure S3. SAXS data measured for the R_3L_{12} at pH 2 dilution series used to incubate DPPG or DPPE liposomes in the dosing assay (inset Figure 5). Concentrations indicated in the plots. The full lines are the fits to the experimental data. The parameters extracted from the fits are listed in Table S2.

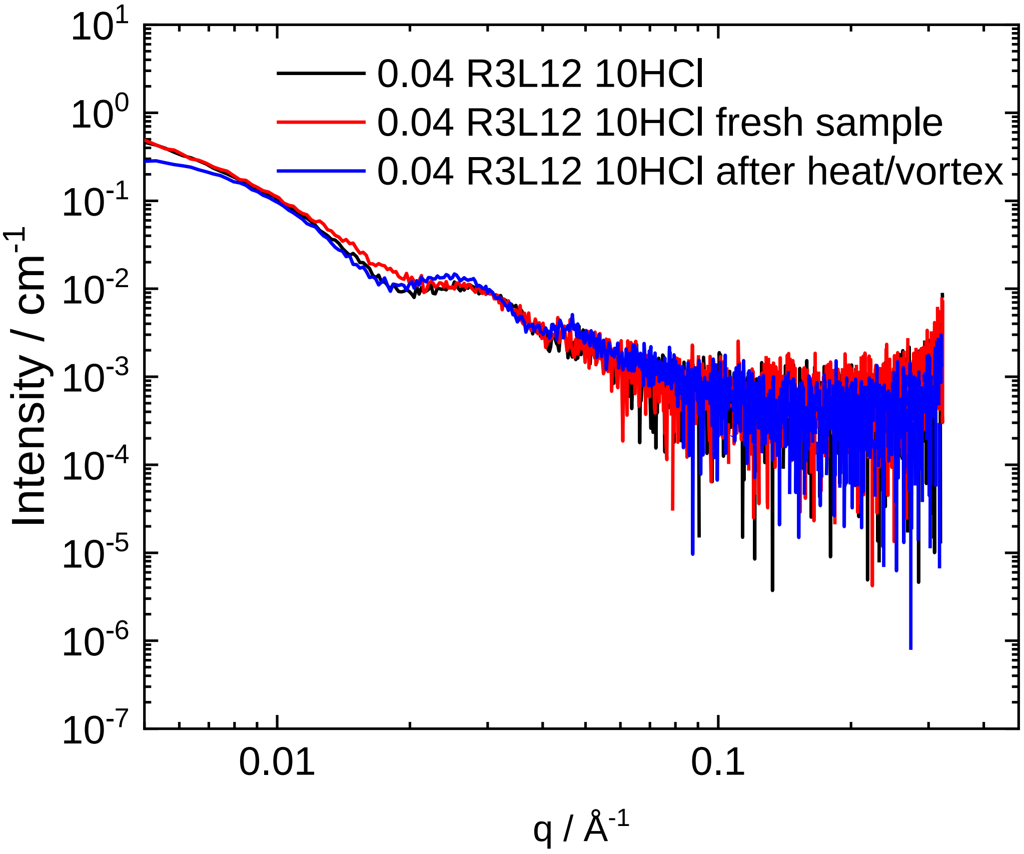

Figure S4. SAXS data measured before and after after heat (65 °C)/vortex treatment (data measured on beamline SWING at synchrotron SOLEIL, France).

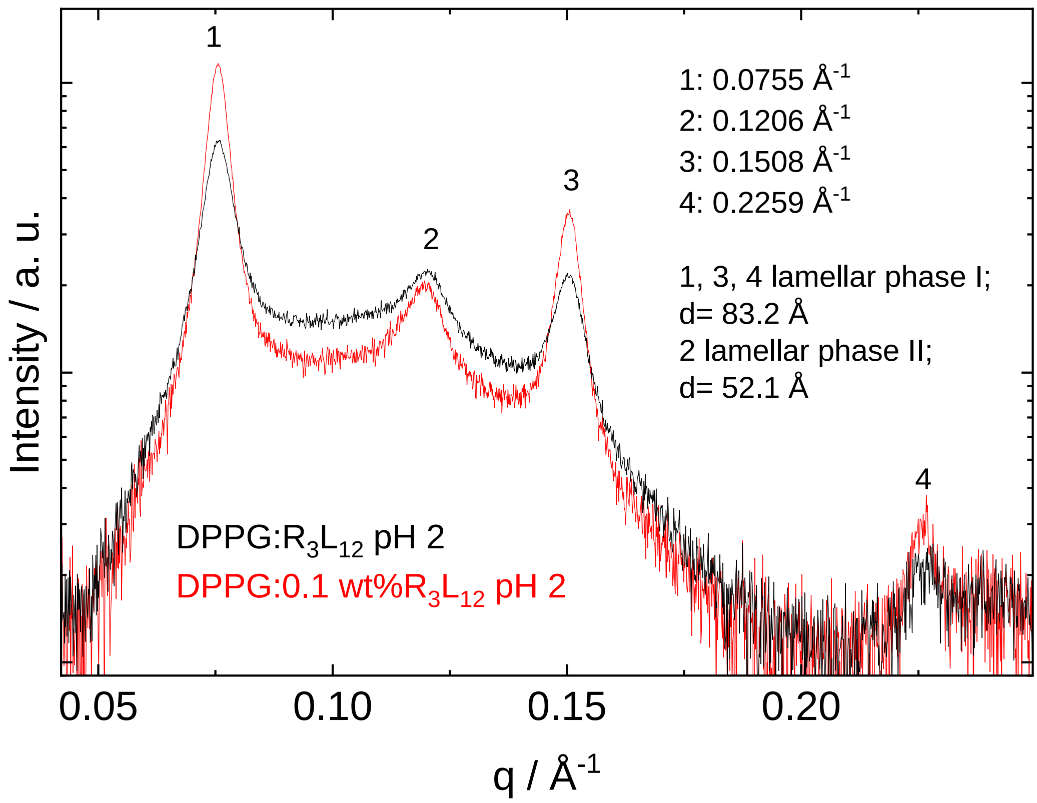

Figure S5. SAXS data for DPPG: R_3L_{12} pH 2, showing the indexation for two lamellar phases with different spacings. Data at 0.1 wt% R_3L_{12} is included to aid the indexation of the peaks. Data for DPPG: R_3L_{12} pH 2 is the same as displayed in Figure 1c.

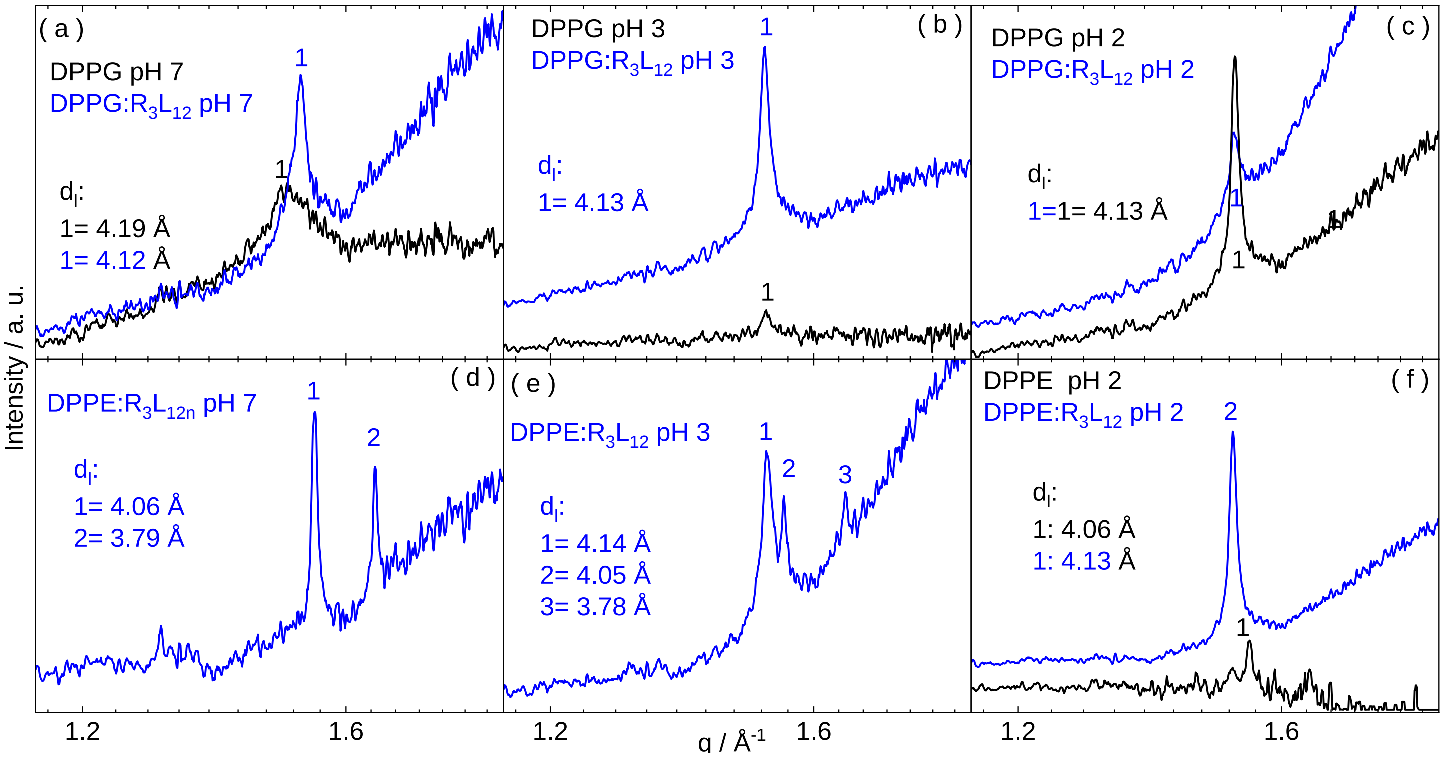

Figure S6. WAXS data for R_3L_{12} or DPPG:R_3L_{12} at (a) pH 7, (b) pH 3, (c) pH 2. WAXS data for DPPE:R_3L_{12} at (d) pH 7, (e) pH 3, (f) R_3L_{12} or DPPG:R_3L_{12} pH 2. Parameter d_l is the lateral spacing between lipid chains.

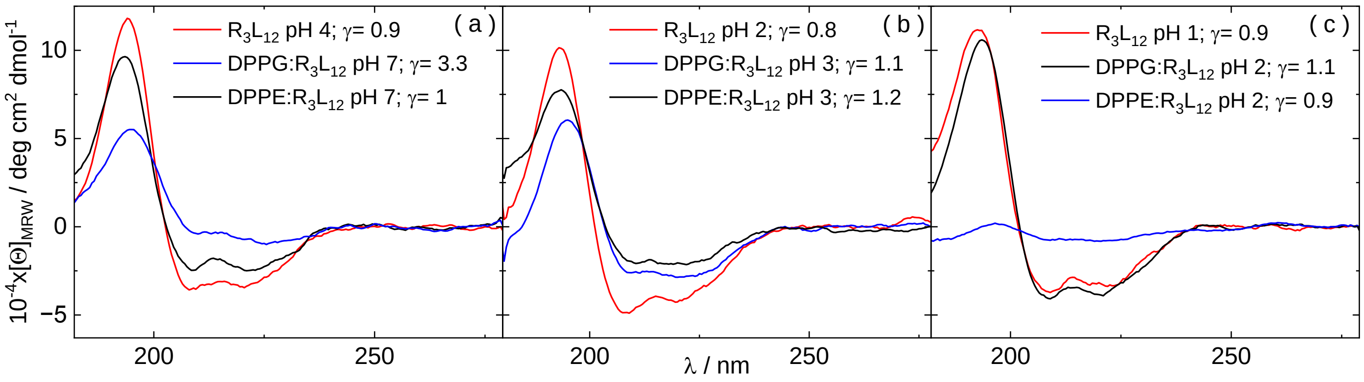

Figure S7. CD spectra for R_3L_{12} at pH (a) 4, (b) 2 or (c) 1 and for DPPG:R_3L_{12} or DPPE:R_3L_{12} at (a) pH 7, (b) pH 3 or (c) pH 2. The values for $\gamma = [\theta]_{222}/[\theta]_{208}$, calculated from the CD curve, are indicated in the figure caption.

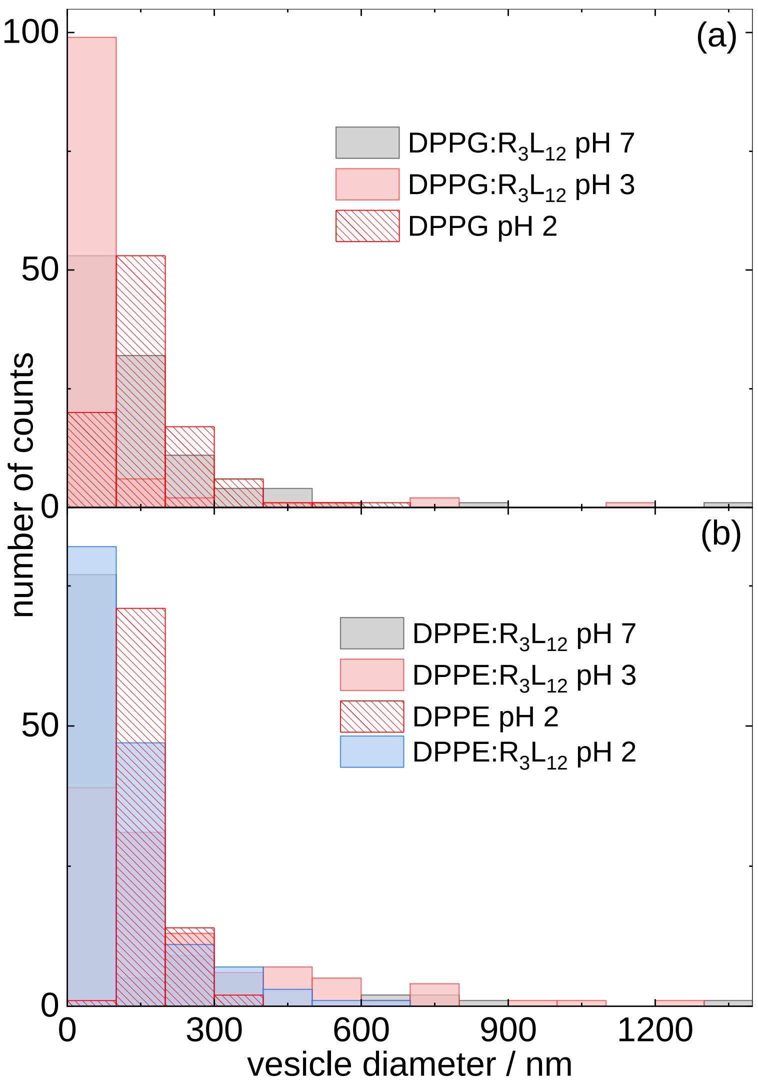

Figure S8. Histograms of vesicle diameters measured from cryo-TEM images for samples containing R_3L_{12} and (a) DPPG or (b) DPPE liposomes.

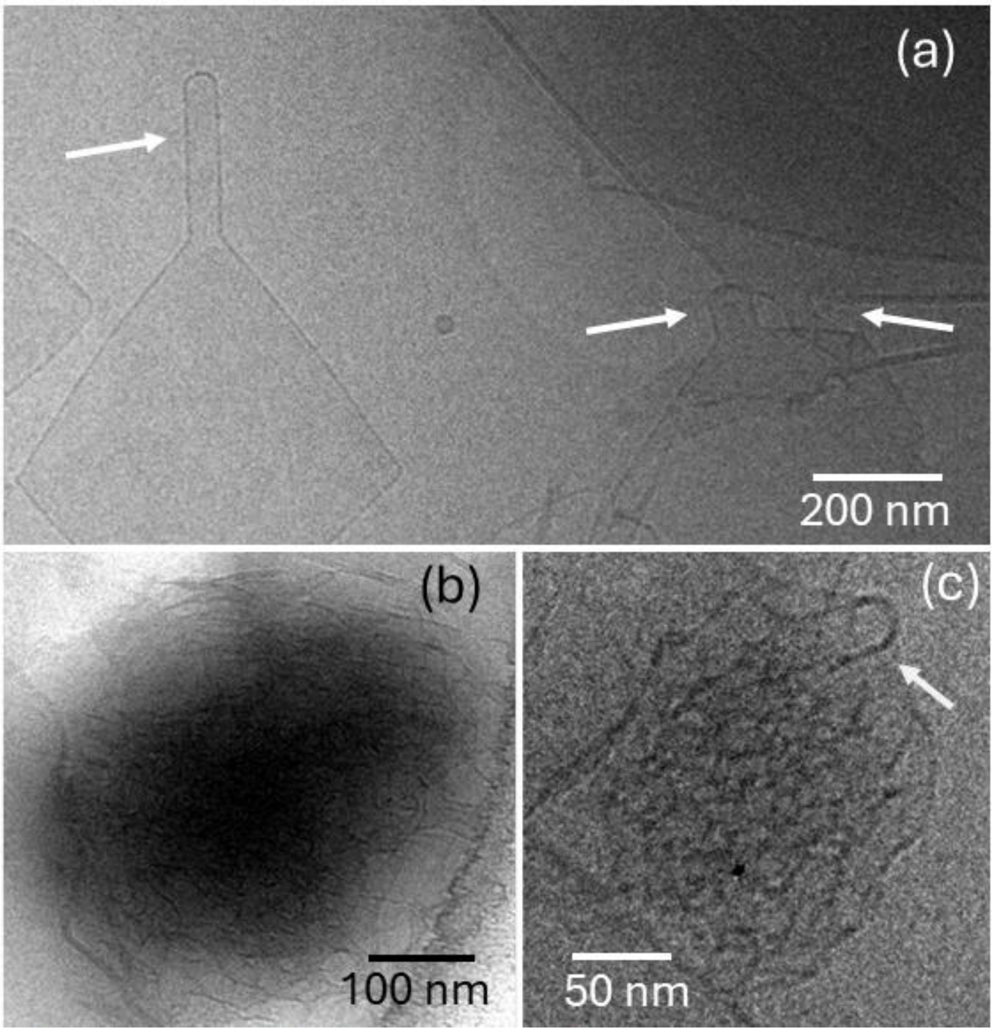

Figure S9. Cryo-TEM images for (a-c) DPPE:R_3L_{12} pH 3; the same sample for which images are displayed in Fig. 3c,d. The arrows in (a) point to nanotubes unfolded into wide sheets. Images in (b-c) suggest that long R_3L_{12} nanotubes can insert in DPPE liposome walls. The arrow in (c) points to a nanotube sticking out of the vesicle wall.

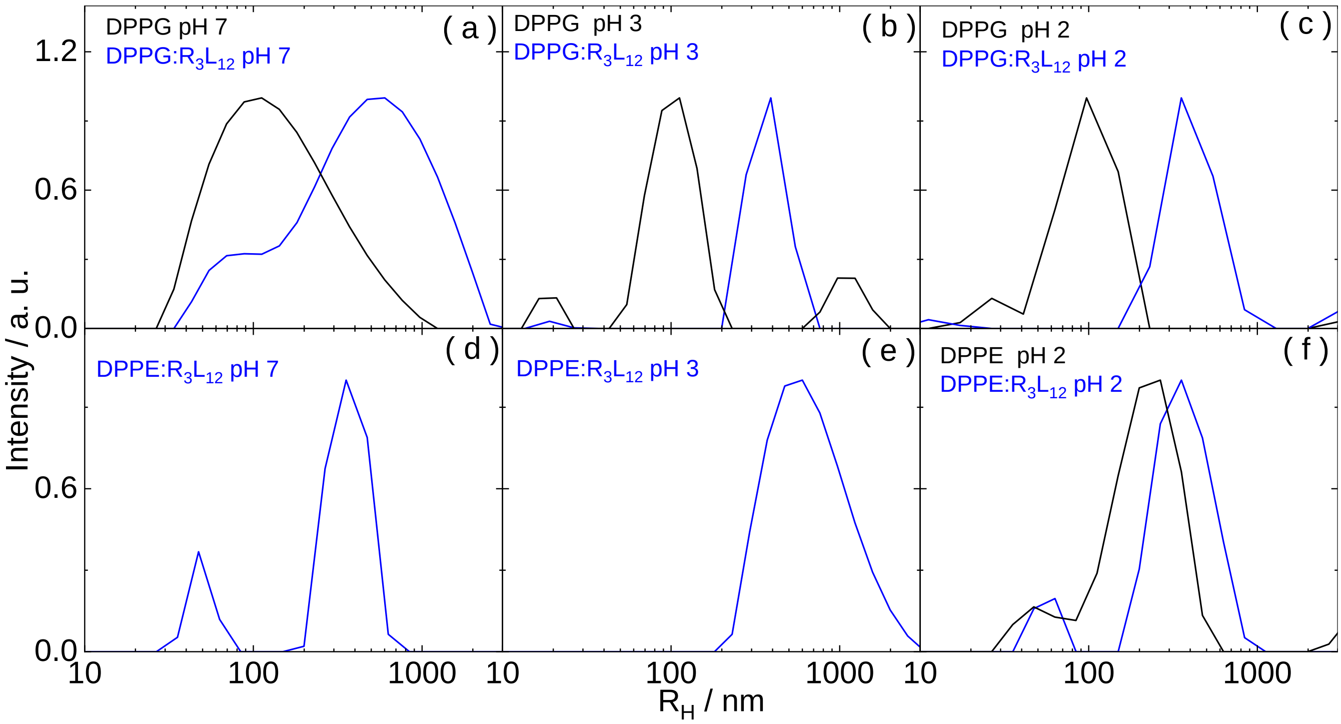

Figure S10. Distributions of hydrodynamic radius from DLS. R_3L_{12} or DPPG:R_3L_{12} at (a) pH 7, (b) pH 3, (c) pH 2. DPPE:R_3L_{12} at (d) pH 7, (e) pH 3, (f) R_3L_{12} or DPPG:R_3L_{12} pH 2.

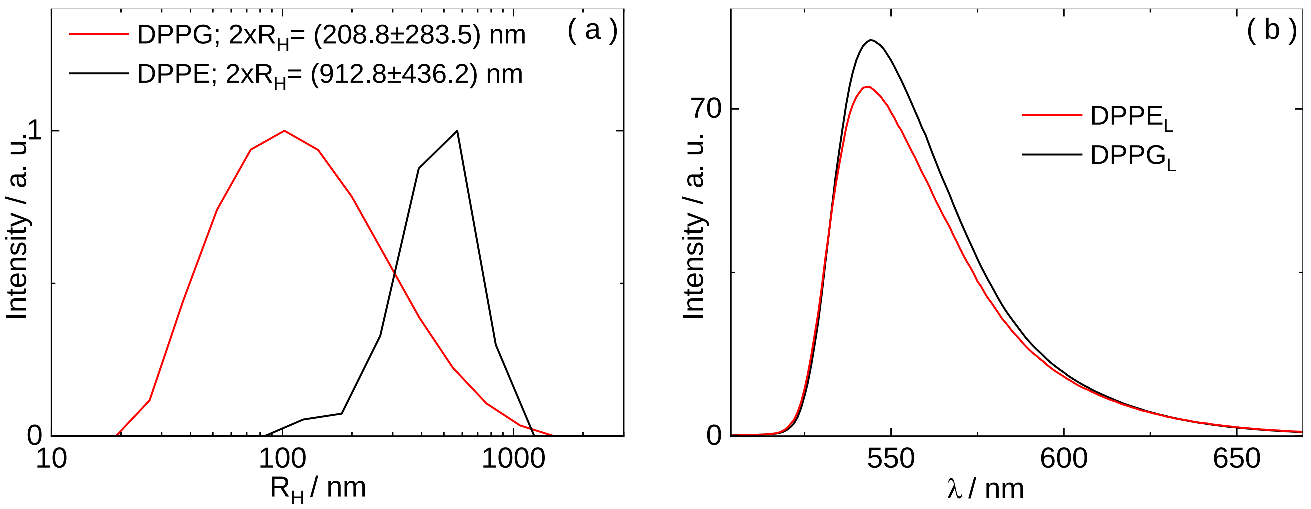

Figure S11. Data for DPPG and DPPE liposomes loaded with calcein and filtered through a Sephadex column: (a) DLS hydrodynamic radius distributions, (b) fluorescence emission curves (λ_{ex}= 485 nm).

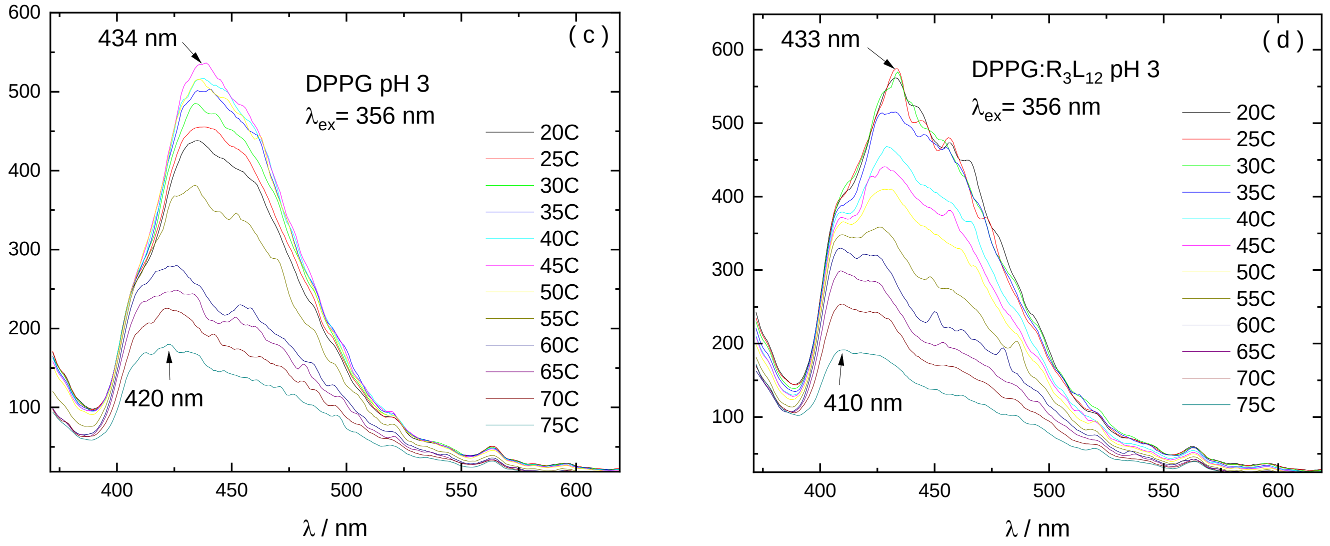

Figure S12. Laurdan fluorescence spectra as a function of the temperature measured for samples containing DPPG. The wavelength for the maximum intensity measured at 20 ^{o}C and 75 ^{o}C, used to calculate the polarization factor, is indicated in the figure caption.

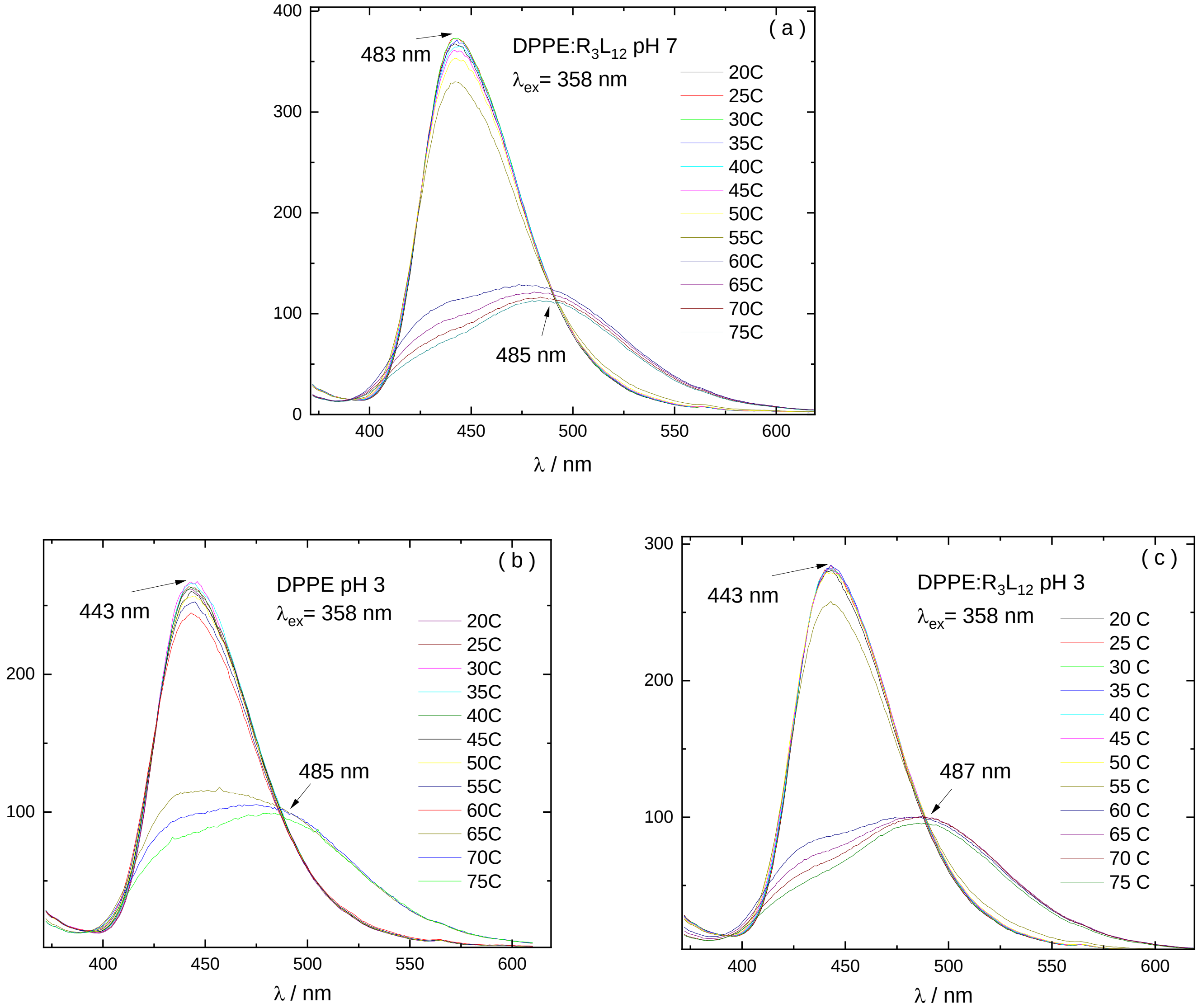

Figure S13. Laurdan fluorescence spectra as a function of the temperature measured for samples containing DPPE. The wavelength for the maximum intensity measured at 20 °C and 75 °C, used to calculate the polarization factor, is indicated in the figure caption.

Table S1. SAXS parameters obtained from the fitting to the SAXS data in Figs S2a-c.

	**R_3L_{12} pH 4**	**R_3L_{12} pH 2**	**R_3L_{12} pH 1**
**N [a.u.]**	1	0.025	0.13
**$R \pm \Delta R$ [Å]**	107±19	124±11	135±20
**D_r [Å]**	21	35	30
**η_{core} [cm^{-1}]**	$3x10^{-10}$	$2.13x10^{-10}$	$1.5x10^{-9}$
**η_{shell} [cm^{-1}]**	$4.5x10^{-8}$	$2.25x10^{-7}$	$0.8x10^{-7}$
**η_{solv} [cm^{-1}]**	$1.46x10^{-9}$	$5.82x10^{-9}$	$7.0x10^{-9}$
**N_1 [a.u.]**	1	3	1.1
**$2z_H \pm \Delta_{2zH}$ [Å]**	25 ± 9	25 ± 10	25 ± 10
**σ_H [Å]**	3.8	2.8	2.5
**η_H [cm^{-1}]**	$-1.13x10^{-7}$	$-1.32x10^{-7}$	$-2.1x10^{-7}$
**σ_C [Å]**	5	3	3
**η_C [cm^{-1}]**	$4.54x10^{-8}$	$8.25x10^{-8}$	$8.252x10^{-8}$
**BG [cm^{-1}]**	$4x10^{-4}$	$7x10^{-4}$	$8x10^{-4}$

Key: ***Cylindrical core-shell:*** scale factor, N; core radius, R; shell thickness, D_r; scattering length density of the core, η_{core}, shell, η_{shell}, and solvent, $\eta_{solv.}$ ***Nanotube wall with Gaussian density profile form factor:*** scale factor, N_1; inter-head group thicknesses, $2z_H$; Gaussian half-width at half-maximum for polydispersity, $\Delta_{2zH,}$;Gaussian half-width for outer layer surface, σ_H; scattering contrast for headgroup, η_H; Gaussian half-width for inner layer, σ_C; scattering contrast for inner layer, η_C; BG background.

Table S2. SAXS parameters obtained from the fitting to the SAXS data in Fig S3.

	**0.02 wt% R_3L_{12} pH 2**	**0.04 wt% R_3L_{12} pH 2***	**0.06 wt% R_3L_{12} pH 2**	**0.08 wt% R_3L_{12} pH 2**	**0.1 wt% R_3L_{12} pH 2**
**N [a.u.]**	0.16	0.025	0.32	0.4	--
**R ± ΔR [Å]**	130±15	124±11	130±15	135±15	--
**D_r [Å]**	18	35	18	18	--
**η_{core} [cm^{-1}]**	$5.7x10^{-10}$	$2.13x10^{-10}$	$5.62x10^{-10}$	$6.12x10^{-7}$	--
**η_{shell} [cm^{-1}]**	$8.5x10^{-8}$	$2.25x10^{-7}$	$8.64x10^{-8}$	$6.89x10^{-7}$	--
**η_{solv} [cm^{-1}]**	$1.6x10^{-9}$	$5.82x10^{-9}$	$1.62x10^{-9}$	$6.1x10^{-7}$	--
**N_1 [a.u.]**	--	--	--	--	1
**R_c±ΔR_c [Å]**	--	--	--	--	59.8±20.6
**ε[cm^{-1}]**	--	--	--	--	$2.57x10^{-8}$
**N_2 [a.u.]**	0.37	3	1.12	1	1
**$2z_H$±Δ_{2zH} [Å]**	25 ± 9	25 ± 10	25 ± 9	25 ± 9	20.6 ± 1.3
**σ_H [Å]**	3.8	2.8	3.8	3.5	3.8
**η_H [cm^{-1}]**	$-1.8x10^{-7}$	$-1.32x10^{-7}$	$-1.8x10^{-7}$	$-2.15x10^{-7}$	$-1.54x10^{-7}$
**σ_C [Å]**	5	3	5	4	5
**η_C [cm^{-1}]**	$4.5x10^{-8}$	$8.25x10^{-8}$	$4.54x10^{-8}$	$4.54x10^{-8}$	$1.91x10^{-8}$
**BG [cm^{-1}]**	$7x10^{-4}$	$7x10^{-4}$	$6x10^{-4}$	$5x10^{-4}$	$5x10^{-4}$

Key: ***Cylindrical core-shell:*** scale factor, N; core radius, R; shell thickness, D_r; scattering length density of the core, η_{core}, shell, η_{shell}, and solvent, $\eta_{solv.}$ ***Long Cylinder:*** scale factor, N_1; cylinder radius, R_c; scattering contrast, ε. ***Nanotube wall with Gaussian density profile form factor:*** scale factor, N_2; inter-head group thicknesses, $2z_H$; Gaussian half-width at half-maximum for polydispersity, $\Delta_{2zH,}$;Gaussian half-width for outer layer surface, σ_H; scattering contrast for headgroup, η_H; Gaussian half-width for inner layer, σ_C; scattering contrast for inner layer, η_C; BG background.

*Same as Table S1

Table S3a. SAXS parameters obtained from the fitting to the SAXS data in Figs 1a-c.

	**DPPG pH 7**	**DPPG:R_3L_{12} pH 7**	**DPPG pH 3**	**DPPG:R_3L_{12} pH 3**	**DPPG pH 2**	**DPPG:R_3L_{12} pH 2**
**N [a.u.]**	1	1	1	1	1	1
**$2z_H \pm \Delta_{2zH}$ [Å]**	26 ± 9	29 ± 10	47± 7	47 ± 7	49 ±6	49 ±6
**σ_H [Å]**	3.5	3.5	2.9	3.5	4.4	4.4
**η_H [cm^{-1}]**	0.9x10^{-6}	0.9x10^{-6}	0.9x10^{-6}	-9x10^{-7}	6.2x10^{-7}	6.2x10^{-7}
**σ_C [Å]**	3.8	3.8	3.8	2.8	4.7	4.7
**η_C [cm^{-1}]**	-0.6x10^{-6}	-0.6x10^{-6}	-0.8x10^{-6}	-9x10^{-7}	-4.2x10^{-7}	-4.2x10^{-7}
**BG [cm^{-1}]**	--	--	5x10^{-3}	8x10^{-4}	6x10^{-4}	6x10^{-4}

Key: ***Gaussian bilayer profile form factor:*** scale factor, N; inter-head group thicknesses, $2z_H$; Gaussian half-width at half-maximum for polydispersity, $\Delta_{2zH,}$;Gaussian half-width for outer layer surface, σ_H; scattering contrast for headgroup, η_H; Gaussian half-width for inner layer, σ_C; scattering contrast for inner layer, η_C; BG, background.

Table S3b. SAXS parameters obtained from the fitting to the SAXS data in Fig 1d-f.

	**DPPE:R_3L_{12} pH 7**	**DPPE:R_3L_{12} pH 3**	**DPPE pH 2**	**DPPE:R_3L_{12} pH 2**
**N [a.u.]**	0.03	0.9	0.5	0.05
**$2z_H \pm \Delta_{2zH}$ [Å]**	50 ± 15	45 ± 10	55 ± 20	42 ± 10
**σ_H [Å]**	0.3	5	3.5	3.5
**η_H [cm^{-1}]**	$1x10^{-5}$	$-1.57x10^{-7}$	$1.9x10^{-7}$	$0.8x10^{-6}$
**σ_C [Å]**	0.5	3.7	3.7	3.8
**η_C [cm^{-1}]**	$-2x10^{-6}$	$1.35x10^{-7}$	$-1.3x10^{-7}$	$-0.7x10^{-6}$
**N_1**	20	8	15	15
**d [Å]**	61	83	61	55.5
**h**	0.2	0.18	0.08	0.08
**n**	2	5	0.05	22
**BG [cm^{-1}]**	0	$5x10^{-4}$	$5x10^{-4}$	0

Key *Gaussian density profile form factor:* scale factor, N; Gaussian half-width at half-maximum for polydispersity, Δ_{2zH}; inter-head group thicknesses, $2z_H$; Gaussian half-width for outer layer surface, σ_H; scattering contrast for headgroup, η_H; Gaussian half-width for inner layer, σ_C, scattering contrast for inner layer, η_C; BG, background. ***Caillé Structure factor:*** number of layers, N_1, stacking separation, d, Caillé parameter, h, and scaling constant, n.

Table S4. Values of hydrodynamic radius, R_H, and zeta-potential, ζ.

Sample	**$2R_H$/ nm**	**$\zeta^{a,b}$/ mV**
DPPG pH 7	212±290	-66.0±0.1
DPPG: R_3L_{12} pH 7	1090±1328	-56.5±0.6
DPPE: R_3L_{12} pH 7	700±270	47.3±1.1
DPPG pH 3	210±89	-67.9±1.2
DPPG: R_3L_{12} pH 3	772±259	-79.8±2.8
DPPE: R_3L_{12} pH 3	1148±914	67.7±1.5
DPPG pH 2	184.8±109.3	-107.8±8.6
DPPG: R_3L_{12} pH 2	673.8±343	-87.0±3.3
DPPE pH 2	452±256	58.1±6.3
DPPE: R_3L_{12} pH 2	718±175	44.4±2.8

[a]Solutions were diluted ×3 to measure ζ.

[b]ζ for 0.013 wt% R_3L_{12} pH 4, pH 2 and pH 1 is 42.9±9.4, 53.6±1.6 and 64.6±7.0 mV respectively.

References

(1) Castelletto, V.; Seitsonen, J.; Ruokolainen, J.; Piras, C.; Cramer, R.; Edwards-Gayle, C. J. C.; Hamley, I. W., Peptide Nanotubes Self-Assembled from Leucine-Rich Alpha Helical Surfactant-Like Peptides. *ChemComm* **2020,** *56*, 11977-11980.
